# Antisense Oligonucleotides Conjugated with Lipophilic Compounds: Synthesis and In Vitro Evaluation of Exon Skipping in Duchenne Muscular Dystrophy

**DOI:** 10.3390/ijms23084270

**Published:** 2022-04-12

**Authors:** Elena Marchesi, Rita Cortesi, Lorenzo Preti, Paola Rimessi, Maddalena Sguizzato, Matteo Bovolenta, Daniela Perrone

**Affiliations:** 1 Department of Environmental and Prevention Sciences, University of Ferrara, 44121 Ferrara, Italy; mrclne@unife.it; 2 Department of Chemical, Pharmaceutical and Agricultural Sciences, University of Ferrara, 44121 Ferrara, Italy; crt@unife.it (R.C.); prtlnz@unife.it (L.P.); sgzmdl@unife.it (M.S.); 3 Unit of Medical Genetics, S. Anna University-Hospital, 44121 Ferrara, Italy; rsp@unife.it; 4 Department of Translational Medicine, University of Ferrara, 44121 Ferrara, Italy

**Keywords:** antisense oligonucleotide, exon skipping, conjugation, 2′-*O*-methyl-phosphorothioate (2′-OMe PS), bile acid, ursodeoxycholic acid (UDCA), lipophilic compound, Duchenne muscular dystrophy

## Abstract

Our groups previously reported that conjugation at 3′-end with ursodeoxycholic acid (UDCA) significantly enhanced in vitro exon skipping properties of ASO 51 oligonucleotide targeting the human DMD exon 51. In this study, we designed a series of lipophilic conjugates of ASO 51, to explore the influence of the lipophilic moiety on exon skipping efficiency. To this end, three bile acids and two fatty acids have been derivatized and/or modified and conjugated to ASO 51 by automatized solid phase synthesis. We measured the melting temperature (*T*_m_) of lipophilic conjugates to evaluate their ability to form a stable duplex with the target RNA. The exon skipping efficiency has been evaluated in myogenic cell lines first in presence of a transfection agent, then in gymnotic conditions on a selection of conjugated ASO 51. In the case of 5′-UDC-ASO 51, we also evaluated the influence of PS content on exon skipping efficiency; we found that it performed better exon skipping with full PS linkages. The more efficient compounds in terms of exon skipping were found to be 5′-UDC- and 5′,3′-bis-UDC-ASO 51.

## 1. Introduction

Duchenne muscular dystrophy (DMD) is a neuromuscular disorder caused by mutations in the dystrophin gene that affect the expression of dystrophin protein. DMD arises because of frame-shifting mutations in the DMD gene encoding dystrophin so that dystrophin protein is absent, leading to fatal muscle degeneration. Lack of dystrophin expression causes progressive wasting of the muscles’ tissues, eventually leading to death as no cure yet exists for this disease [1,2].

Therapeutic approaches aimed at restoring a functional dystrophin gene require either correction or replacement of the mutated gene [3,4]. One of the most promising therapeutic strategies for the treatment of neuromuscular disorders is based on the use of antisense oligonucleotides (ASO) able to effect splice correction of aberrant pre-mRNA. They recognize a specific sequence by nucleic acid base-pairing and modulate the splicing of out-of-frame pre-mRNA leading to restoration of the reading frame encoding for a truncated but functional Becker-like dystrophin protein [5]. A variety of ASO chemistries designed for splicing modulation have been tested in pre-clinical and clinical [3,6,7,8,9,10,11]. Nevertheless, only few modifications have received FDA approval to treat genetic disorders by using splicing modifier ASO. Specifically, the phosphorodiamidate morpholino oligomers (PMO) viltolarsen, eteplirsen, golodirsen, casimersen for the treatment of DMD and the 2′-*O*-methoxyethyl-phosphorothioate (2′-MOE PS) oligomers nusinersen and milasen to treat spinal muscular atrophy (SMA) and Batten disease, respectively [12,13,14,15]. Although encouraging results have been obtained in most cases, certain limitations of current ASO chemistries, such as poor tissue uptake, remain to be solved [16,17].

Other chemistries have been developed to improve potency, bioavailability, and safety of ASO. Recently, Goyenvalle et al. demonstrated that tricycle-DNA (tc-DNA) ASO targeting the exon 51 of human DMD gene, displayed unprecedented uptake in many tissues after systemic administration in vivo in transgenic hDMD and mdx52 mouse models [7,18,19,20].

In addition, other studies have focused on the modification of well-studied ASO to enhance their delivery and improve their physicochemical properties. To this end, the conjugation to appropriate small molecules represents an attractive strategy for improving therapeutic properties of ASO [21]. For example, a newer generation of PMO conjugated with cell-penetrating peptides (PPMO) has proven positive clinical results from a phase II trial with DMD patients, in terms of dystrophin restoration with respect to the naked analogue sequence [22]. A preclinical study in dystrophic mouse has reported that conjugation to palmitic acid enhanced the potency of tc-DNA ASO in both skeletal and cardiac muscles [23]. Among the various functional small molecules for conjugations, neutral lipids, such as fatty acids, cholesterol, or squalene have attracted considerable interest due to their ability to enhance uptake and activity of ASO in certain tissues, including skeletal and cardiac muscle in mice [17,24,25,26].

**Figure 1:**
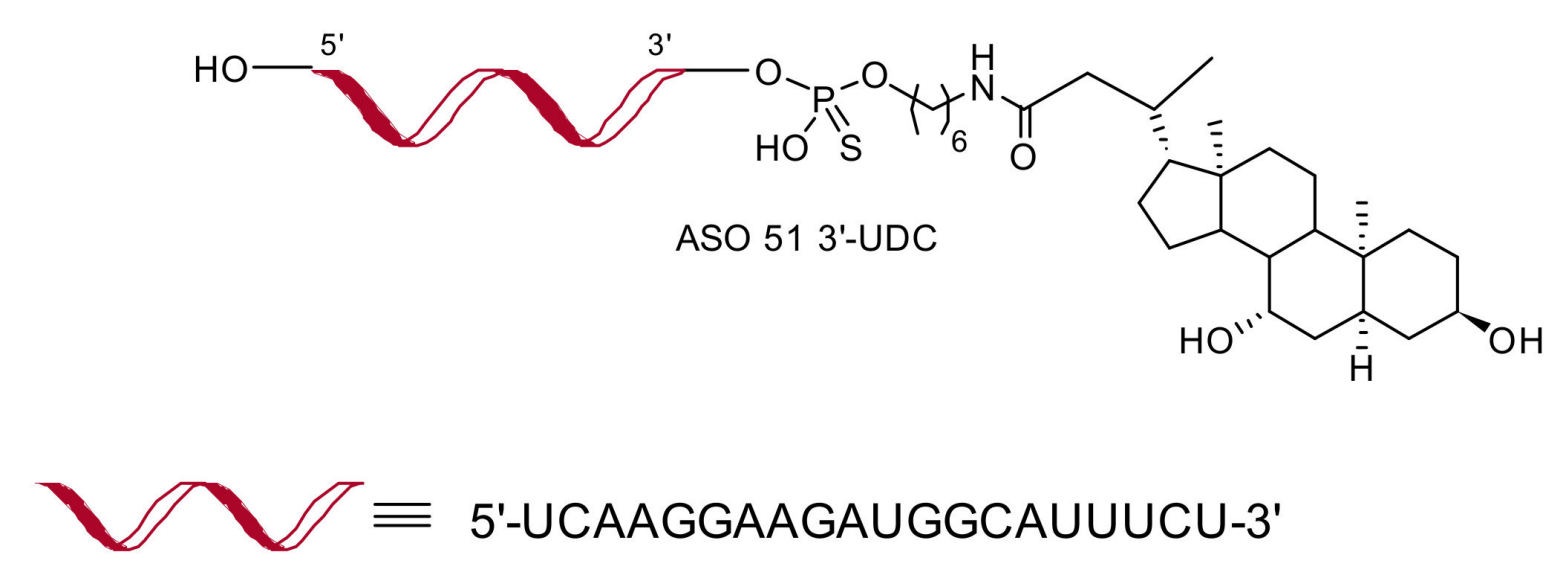
Chemical structures of ASO 51 3′-UDC previously reported.

Recently, we reported for the first time the synthesis and in vitro exon skipping properties of ASO 51 conjugated at 3′-end with ursodeoxycholic acid (ASO 51 3′-UDC, Figure 1) [27]. ASO 51 is a 20-mer RNA-based oligonucleotide full-length modified as 2′-OMe PS, containing the sequence of clinically evaluated Drisapersen, targeting the human DMD exon 51 [6,28,29]. We observed no toxicity and a significant enhancement of the targeted exon 51 skipping when compared with naked ASO 51 in human immortalized myogenic cells. We assumed that amphiphilic nature of UDCA together with its anti-apoptotic properties could be beneficial for the activity of ASO 51 [30,31].

**Figure 2:**
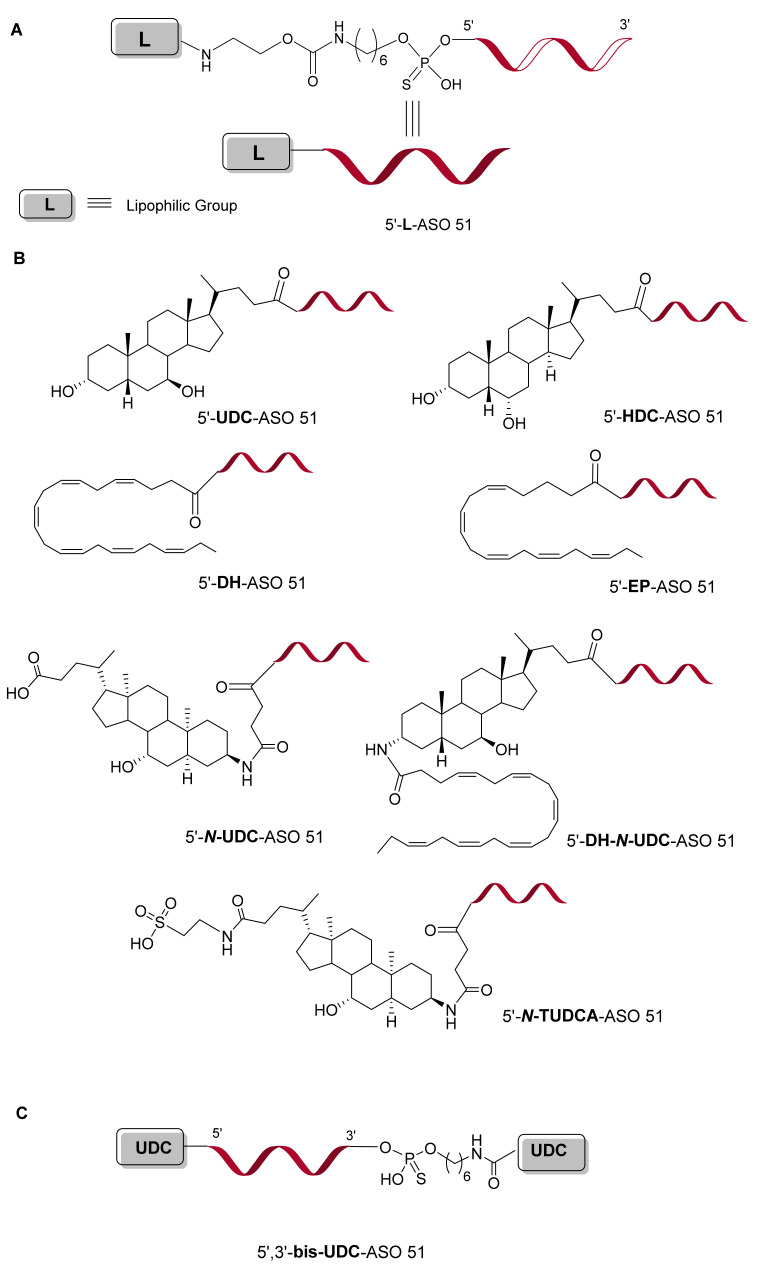
Chemical structures of lipophilic-modified ASO 51 synthesized in this work. (**A**) Structure of general ASO 51 conjugated at 5′-end with a lipophilic compound (**L**). (**B**) Structures of 5′-**L**-ASO 51. (**C**) Structure of 5′,3′-bis-UDC ASO 51.

The exciting results prompted us to design a library of ASO 51 modified with different lipophilic groups to explore the influence of the lipophilic moiety on exon skipping efficiency. For this purpose, a selection of lipophilic moieties was prepared and conjugated at 5′-end achieving a collection of new ASO 51 (5′-L-ASO 51, Figure 2A,B). Particularly, in this study we selected ursodeoxycholic acid (UDCA), hyodeoxycholic acid (HDCA), and tauroursodeoxycholic acid (TUDCA) as model bile acids because of their peculiar physicochemical and biochemical profile [32,33]; and we selected docosahexaenoic acid (DHA) and eicosapentaenoic acid (EPA), two omega-3 fatty acids with beneficial effects on human health [34,35,36]. Several derivatives of the above compounds were prepared in our laboratories and utilized for conjugation to achieve the lipophilic-modified ASO 51 depicted in Figure 2B. Furthermore, ASO 51 linked to two residues of UDCA, one for each end was also considered (Figure 2C). Finally, we also aimed to evaluate the influence of PS content on exon skipping efficiency of 5′-UDC-ASO 51. To this end, several 5′-UDC-ASO targeting the human DMD exon 51 and carrying different amounts of PS linkages (32, 42, 53 and 68%) were synthesized.

We describe herein the preparation of lipophilic groups and their conjugation to ASO 51 in solid phase conditions. Melting temperature (*T*_m_), photon correlation spectroscopy (PCS), and transmission electron microscopy (TEM) analyses were carried out to obtain an insight into the thermal stability of duplexes with complementary RNA and preferred conformation of selected L-ASO 51. Then, exon skipping efficiency studies in human immortalized myogenic cells were carried out both with and without chemical transfection reagents, in order to explore the influence of lipophilic moieties. In the last case, the so-called gymnotic uptake has been investigated on lipophilic conjugates showing a better exon skipping efficiency.

## 2. Results and Discussion

### 2.1. Design and Synthesis of ASO 51 Conjugated with Lipophilic Compounds

In our previous paper, we reported on the synthesis and exon skipping properties of ASO 51 conjugated at 3*′*-end with UDCA involved in the targeting of human DMD exon 51. ASO 51 3*′*-UDC was successfully prepared by us, exploiting two different approaches and was characterized by good ability to form a stable duplex with its complementary RNA, no toxicity in human immortalized myogenic cells and a better exon skipping efficiency respect to naked ASO 51. UDCA was selected due to certain positive features as for instance, its long-established safety profile [37] and its amphiphilic character [31] that could improve the physicochemical properties and then the pharmacokinetic of our ASO conjugates.

Having clear evidence of increased ability of ASO 51 3*′*-UDC to induce exon skipping when compared to naked one, we designed the synthesis of a series of ASO 51 conjugated at 5*′*-end with a selection of lipophilic compounds (Figure 2B, C) to obtain an insight into their effects.

To attach the lipophilic moieties to the 5*′*-end of ASO 51 we have chosen a solid phase approach based on the formation of the amide bond that plays a pivotal role in the composition of biological systems and shows a good stability during the deprotection/cleavage operations involved in ASO synthesis [38]. Typically, the conjugation of small molecules to oligonucleotides is performed in solution (the so-called post-synthetic conjugation approach), by exploiting well-established protocols [39,40]. Nevertheless, certain small molecules, such as the lipophilic ones, can limit conjugation yields due to their poor solubility in aqueous solutions conditions [40]. By contrast, conjugation can be performed with oligonucleotides held onto the solid supports used for automated solid-phase synthesis (namely pre-synthetic conjugation approach). The major advantage in using a solid-phase approach results in less laborious purifications: the unreacted reagents generally used in considerable excess to achieve high coupling efficiency, can be removed by simple flushing of solid support.

The linking of lipophilic moieties to the 5*′*-end of ASO 51 by amide bond required an amine group in one component and a carboxylic group in the other. To this purpose, in this work ASO 51 was armed with the (aminoethoxycarbonyl)aminohexyl linker (ssH-Linker^TM^) and the lipophilic compounds were converted into *N*-hydroxysuccinimide (NHS) esters or condensed by the aid of a coupling reagent.

#### 2.1.1. Conversion of UDCA, HDCA, DHA, and EPA into Their *N*-Hydroxysuccinimide Esters

**Scheme 1:**
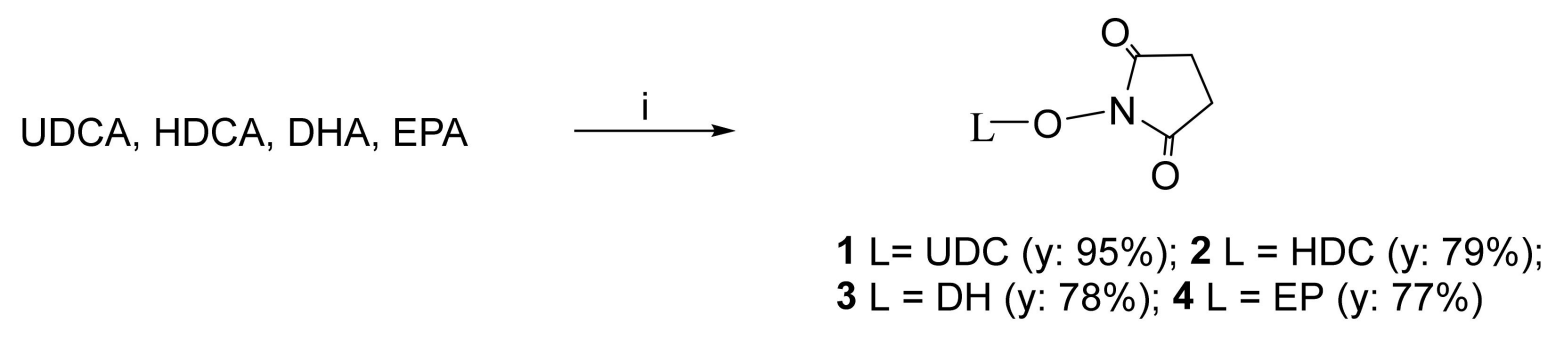
Preparation of NHS esters **1**–**4**. (i) NHS (1.2 eq), DCC (1.2 eq), THF; 25 °C, 18 h.

UDCA, HDCA, DHA, and EPA were converted into their *N*-hydroxysuccinimide (NHS) esters **1**–**4** (Scheme 1), according to the method previously described by us [27]. Esters **1**–**2** were purified by crystallization, on the other hand, esters **3**–**4** containing DHA and EPA acyl residues required a purification by flash chromatography.

#### 2.1.2. Preparation of NHS Esters 8 and 10, and of 3-α-Amino TUDCA 12 via the Common Intermediate 3-α-Amino UDCMe 6

**Scheme 2:**
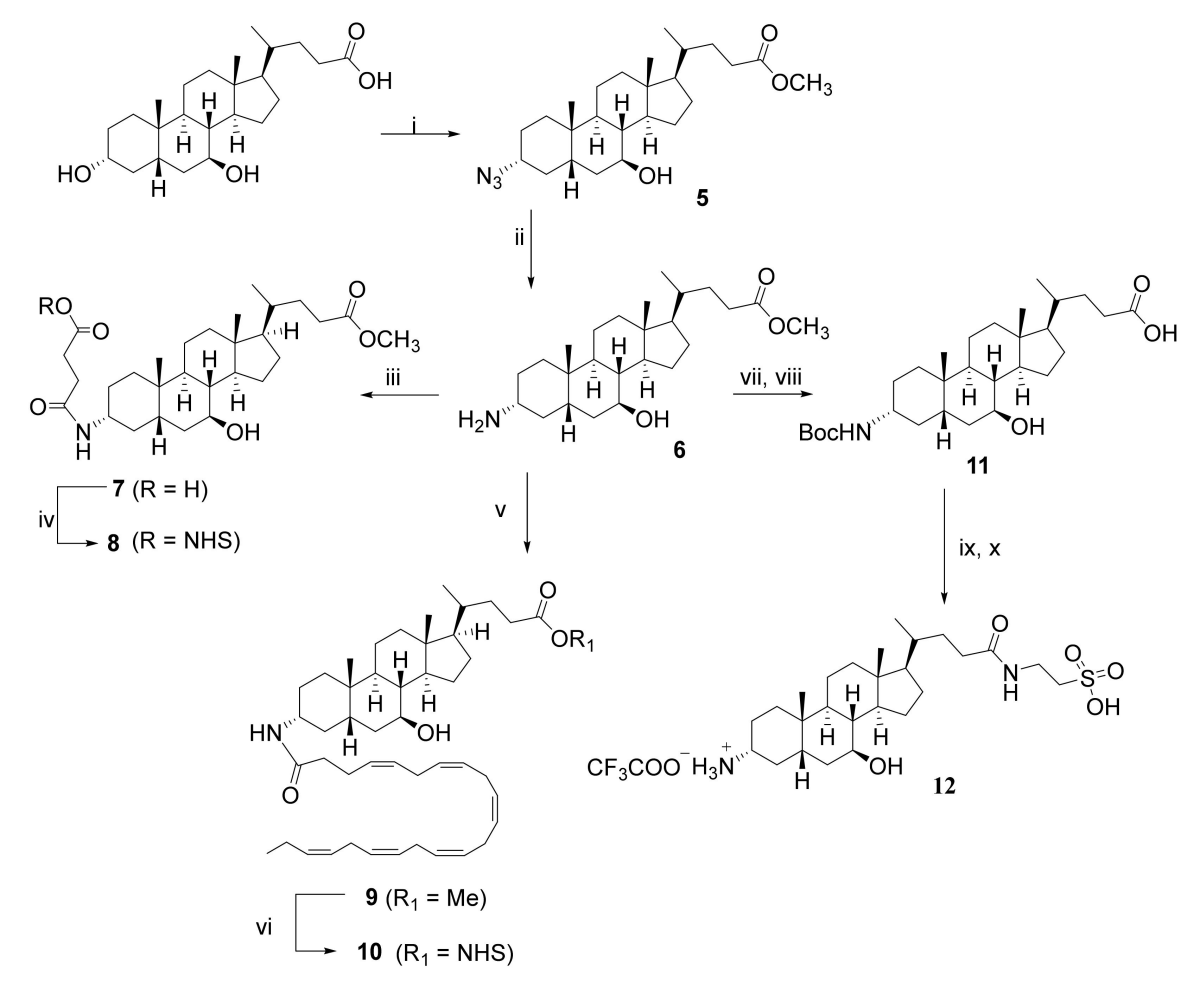
Synthesis of **8**, **10**, and **12** via the common intermediate 3-α-amino UDCMe **6**. (i) [41]; (ii) HCOONH_4_, Pd/C, AcOEt, MeOH, 70 °C, 18 h (78% yield); (iii) succinic anhydride, DMAP, pyridine, 115 °C, 18h; (iv) NHS, N,N′-dicyclohexylcarbodiimide (DCC), THF, 25 °C, 18 h (39% yield from **6**); (v) **3**, DIPEA, DMF, 25 °C, 18 h (65% yield); (vi) LiOH, H_2_O, MeOH, 25 °C, 18 h, then NHS, DCC, THF, 25 °C, 18 h (43% yield); (vii) (BOC)_2_O, NaHCO_3_, THF, 25 °C, 18 h (80% yield); (viii) LiOH, H_2_O, CH_3_OH, 25 °C, 12 h; (ix) ethylchloroformate, TEA, THF, 25 °C, 1 h, then taurine, NaOH, H_2_O, 25 °C, 24 h; (x) trifluoroacetic acid (TFA), CH_2_Cl_2_, 0 °C, 30 min (32% yield from **6**).

Compounds **8**, **10**, and **12**, could be considered as close derivatives of UDCA since they all contained its 3-α-amino analogue **6**. Their synthetic approach is depicted in Scheme 2. It considered the reduction of 3-α-azide **5**, synthesized by us [41], into 3-α-amine **6.** The reaction took place in 18 h at 70 °C, in the presence of ammonium formate (HCOONH_4_) and Pd/C as a catalyst. Compound **6** was obtained in good yield and was used for the preparation of lipophilic compounds **8**, **10**, and **12** without any purification. To prepare NHS ester **8**, 3-α-amine **6** was reacted first with succinic anhydride in the presence of catalytic 4-(dimethylamino)pyridine (DMAP), then, crude acid **7** was converted to NHS ester **8** in usual conditions. The reaction of 3-α-amine **6** with NHS ester **3** and *N,N*-diisopropylethylamine (DIPEA) afforded the intermediate ester **9**, which was converted into NHS-ester **10** after cleavage of methyl ester with aqueous LiOH in methanol and reaction of the resulting acid with *N*-hydroxysuccinimide in usual conditions. Finally, to achieve 3-α-amino TUDCA **12** in satisfactory yield and purity, the free amino group of compound **6** was subjected to a protection-deprotection strategy that allowed for a selective introduction of amino sulfonic acid taurine.

It is worth noting that, the conversion of amine **6** into 3-α-amino TUDCA **12,** required only one purification by flash chromatography after BOC addition and crude **12** was used for conjugation to ASO 51 without any purification.

#### 2.1.3. Solid-Phase Synthesis of Lipophilic-Modified ASO 51

**Scheme 3:**
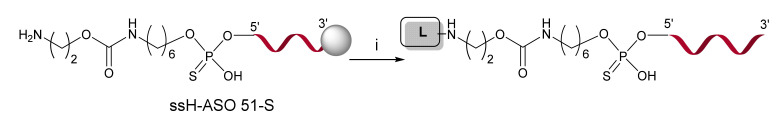
Solid-phase synthesis of 5′-L-ASO 51. (i) See Table S1 for reagents and conditions.

**Scheme 4:**
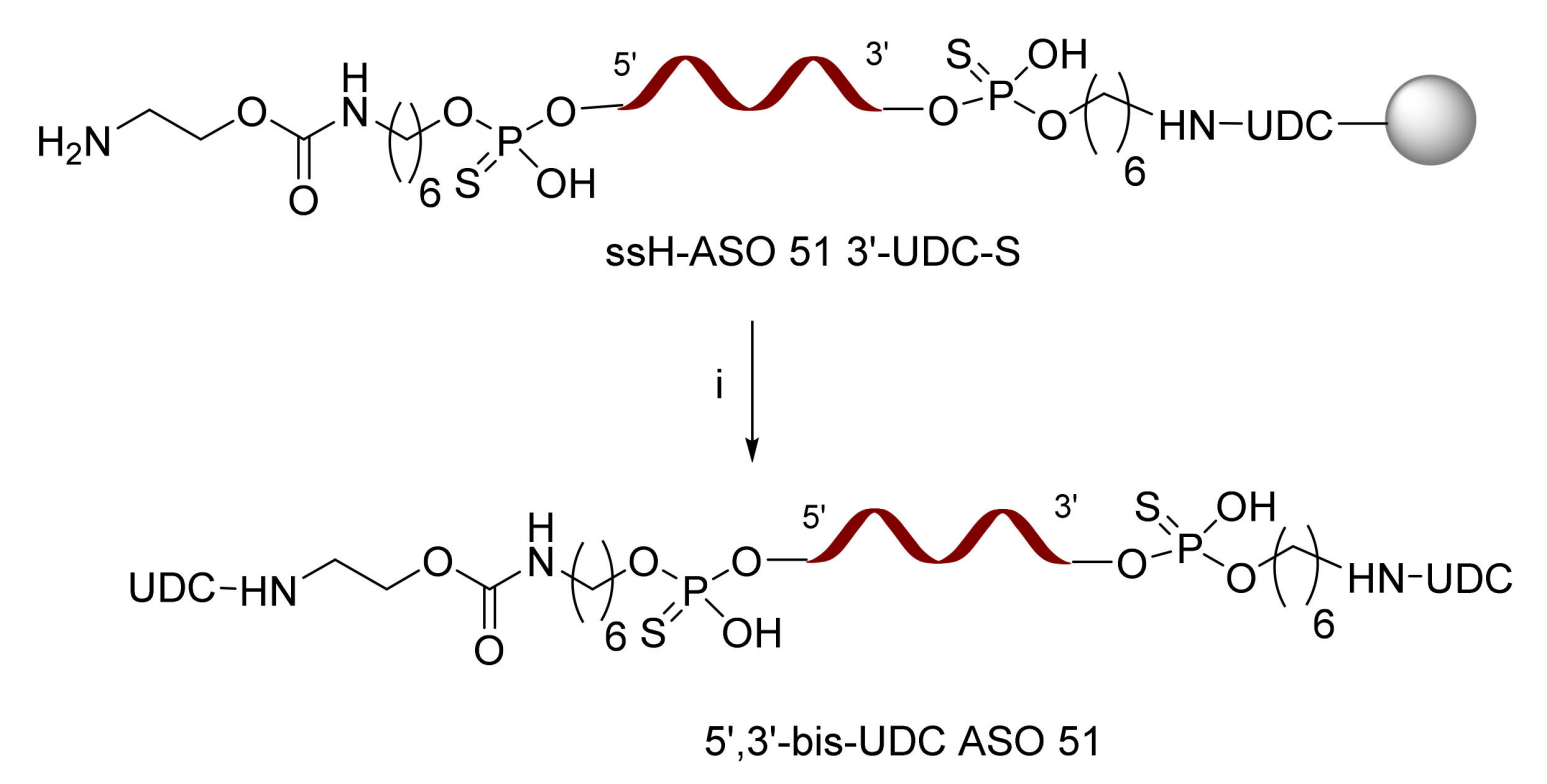
Solid-phase synthesis of 5′,3′-bis-UDC-ASO 51. (i) See Table S1 for reagents and conditions.

In this work, we prepared and characterized several ASO 51 modified with a selection of lipophilic moieties using the solid-phase phosphoramidite chemistry. Our conjugation approach involved the coupling reaction between a free amino group presented onto the solid-support and the carboxylic group of target molecules. To this end, the ssH-Linker^TM^ protected as monomethoxytrityl (MMT), was inserted at 5′-end of ASO 51 after addition of the last nucleotide of the sequence, exploiting the phosphoramidite chemistry. The fast deprotection of MMT group [42] allowed a straightforward solid-phase conjugation of ssH-ASO 51-S with target lipophilic derivatives, as reported in Scheme 3 and Table S1. ASO 51 linked to UDC (5′-UDC-ASO 51), HDC (5′-HDC-ASO 51), DH (5′-DH-ASO 51), EP (5′-EP-ASO 51), *N*-UDC (5′-*N*-UDC-ASO 51), and DH-*N*-UDC (5′-DH-*N*-UDC-ASO 51) were synthesized by using the prepared NHS-ester **1**–**4**, **8,** and **10**, **respectively**. To synthesized 5′-*N*-TUDCA-ASO 51, the free amino group of ssH-ASO 51-S was armed with a succinic acid linker before adding *N*-TUDCA derivative **12** in presence of 2-(1*H*-benzotriazol-1-yl)-1,1,3,3-tetramethyluronium hexafluorophosphate (HBTU) as a coupling reagent. The synthesis of 5′,3′-bis-UDC-ASO 51, in which two residues of UDCA were added one for each end of ASO 51, was achieved by using our ASO 51 3′-UDC prepared via the pre-synthetic approach previously reported which required the synthesis of a novel solid support functionalized with UDCA before assembling the oligonucleotide [27]. Thus, as depicted in Scheme 4, ASO 51 3′-UDC held on to solid support was converted into the desired 5′,3′-bis-UDC-ASO 51 in the same conditions employed for 5′-UDC-ASO 51.

After conjugations, cleavage from solid support and protective groups removal in standard ammonia conditions, afforded desired L-ASO 51 conjugates. The efficiency of solid-phase conjugations ranging from 15 to 83%, was estimated from UV detection of ion-pair HPLC (IP-HPLC) analysis (Table S1).

Finally, all crude L-ASO 51 were first purified by IP-HPLC with a triethylammonium acetate (TEAA) buffer and then by ion exchange HPLC (IEX-HPLC) for sodium exchange. The molecular masses were confirmed by ESI-MS analysis (Table S1).

#### 2.1.4. Synthesis of 5′-UDC-ASO Carrying Different Amounts of PS Linkages

To evaluate the effect of PS content on the exon skipping efficiency of the 5′-UDC-ASO targeting the human DMD exon 51, we designed the ASO with different amounts of PS linkages detailed in Table 1. Conjugation with UDC at 5′-end of ASO and purification of final compounds were carried out as for 5′-UDC-ASO 51. The molecular mass performed by ESI-MS analysis confirmed the correct content of PS linkages (see Table S1).

### 2.2. Lipophilic-Modified ASO 51 Formed Stable Duplex with Their Complementary RNA

The melting temperatures (*T*_m_) of the duplex formed by lipophilic ASO 51 with the natural RNA complementary sequence were measured and compared with the corresponding duplex formed by naked ASO 51 and they are summarized in Table 2. From these data, it appeared that single lipophilic moieties at 5′-end of ASO 51 did not destabilize the duplex, on the contrary to 5′-DH-*N*-UDC-ASO 51 in which two different lipophilic compounds were incorporated (Δ*T*_m_ of −3.52 °C). Finally, also the addition of two residues of UDCA, one for each end, as in the case of 5′,3′-bis-UDC-ASO 51 enabled the formation of a stable duplex.

### 2.3. In Vitro Tests of ASO 51 Conjugated with Lipophilic Compounds

#### 2.3.1. 5′-UDC Conjugation Further Increases Potency of ASO 51 Compared to 3′-UDC

**Figure 3:**
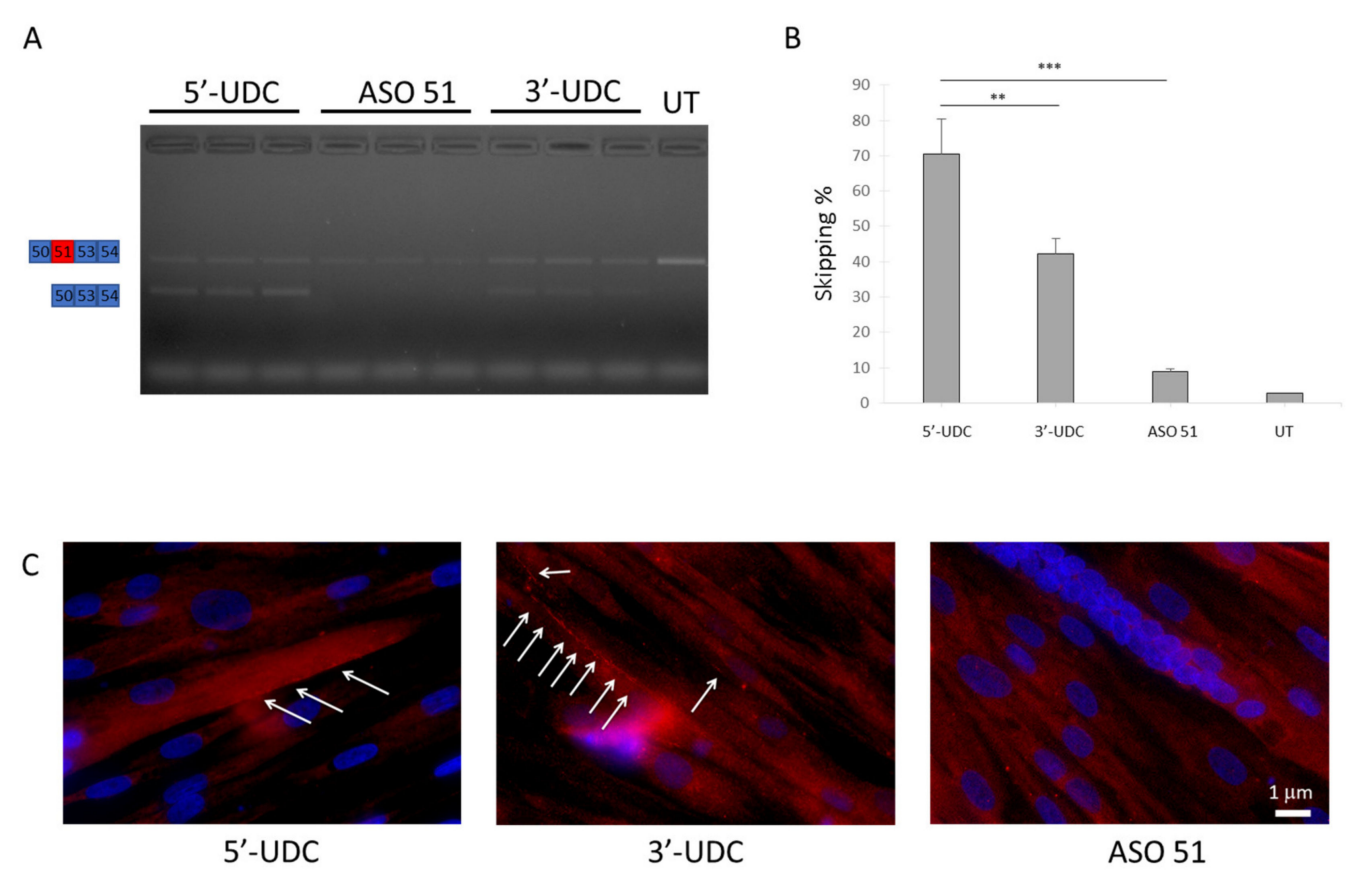
Exon skipping analysis of 5′-UDC versus 3′-UDC and ASO 51 in human myogenic cells bearing a deletion of the DMD exon 52. (**A**) 28 cycles RT-PCRs performed with primers DMDex50F and DMDex54R amplifying the dystrophin transcript and the skipped isoform. Then, 1 uL of each RT-PCR was loaded onto the high-sensitivity DNA Chip and the 2100 Bioanalyser for quantification of the exon skipping efficiency reported in (**B**). Results are expressed as mean ± SEM; *n* = 3 tests per group. *** *p* < 0.001 and ** *p* < 0.01 (paired *t*-test with post hoc Mann–Whitney test). UT = Untreated. (**C**) Immunocytochemistry of dystrophin (red) and nuclei (blue) in myotubes from patient with exon 52 deletion treated with 5′-UDC, 3′-UDC, or ASO 51. White arrows show the presence of dystrophin at the inner face of the plasma membrane. Scale bar, 1 μm.

We have previously demonstrated that ASO 51 conjugated at 3′-end with UDCA perform significantly better than ASO 51 to skip exon 51 in human myoblasts. To further explore the properties of the UDCA conjugation we tested the 5′-UDC-ASO 51 in comparison with the 3′ one and the unconjugated ASO 51 in myotubes obtained upon differentiation from immortalized myoblasts bearing a deletion of exon 52 (Figure 3).

Exon skipping efficiencies resulted of about 70% for 5′-UDC conjugated ASO, ~45% for ASO 51 3′-UDC and nearly 8% for ASO 51 prompting the 5′-UDC-ASO 51 as the significantly more efficient ASO in this round of experiments.

#### 2.3.2. Evaluation of the Variable PS Content in the 5′-UDC-ASO 51

**Figure 4:**
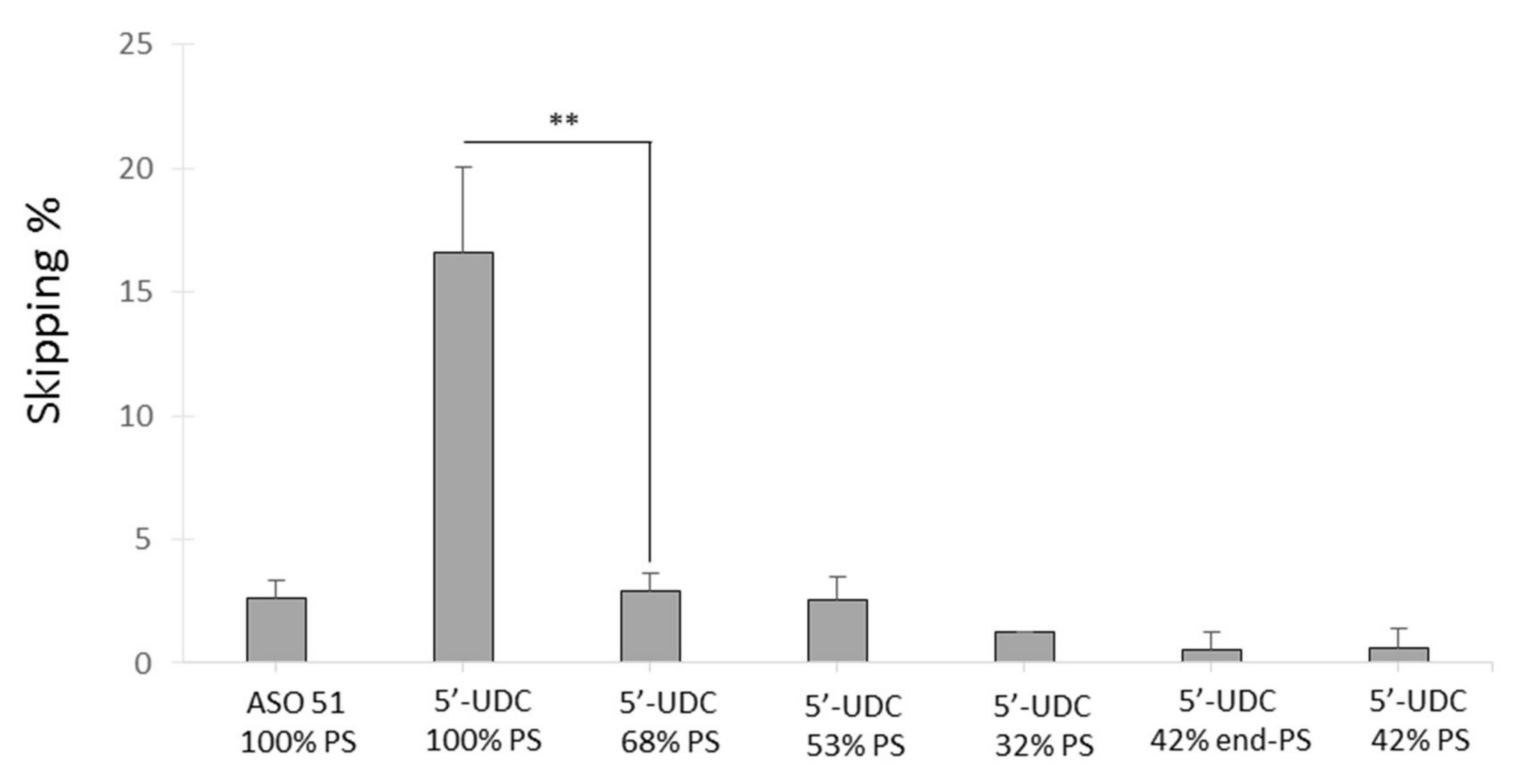
Exon skipping analysis ASO 51 and 5**′**-UDC-ASO 51 with different amounts of PS linkages. Results are expressed as mean ± SEM; *n* = 3 tests per group. ** *p* < 0.01 (paired *t*-test with post hoc Mann–Whitney test).

After establishing 5′-UDC conjugated ASO 51 as the most efficient compound (8.75 fold increase respect to ASO 51) we explored the possibility to reduce PS linkages, minimizing PS associated toxicity, without compromising exon skipping efficacy. The 5′UDC-ASO 51 with different PS content (Table 1) have been synthetized and transfected in Δ52 myotubes for testing the skipping efficiency. Overall, 68% of PS linkages caused a significant reduction in exon skipping efficiencies to a level like that of the ASO 51. All other oligos with reduced PS content had similar or lower levels of skipping regardless the distribution of the PS linkages (Figure 4). Overall, these results indicates that UDC conjugation may allow the reduction in PS content to reach the same or higher levels of skipping than ASO 51, but the best results are obtained with full PS linkages. Further studies will be needed to elucidate whether a reduction in PS content in the 5′-UDC-ASO 51 reflect an improved safety profile of this ASO, but some conclusions can be drawn considering that similar results have been seen in the extended studies performed with tc-DNA ASO with reduced PS contents [43].

#### 2.3.3. Evaluation of Variable Lipophilic Compounds Conjugated to ASO 51

**Figure 5:**
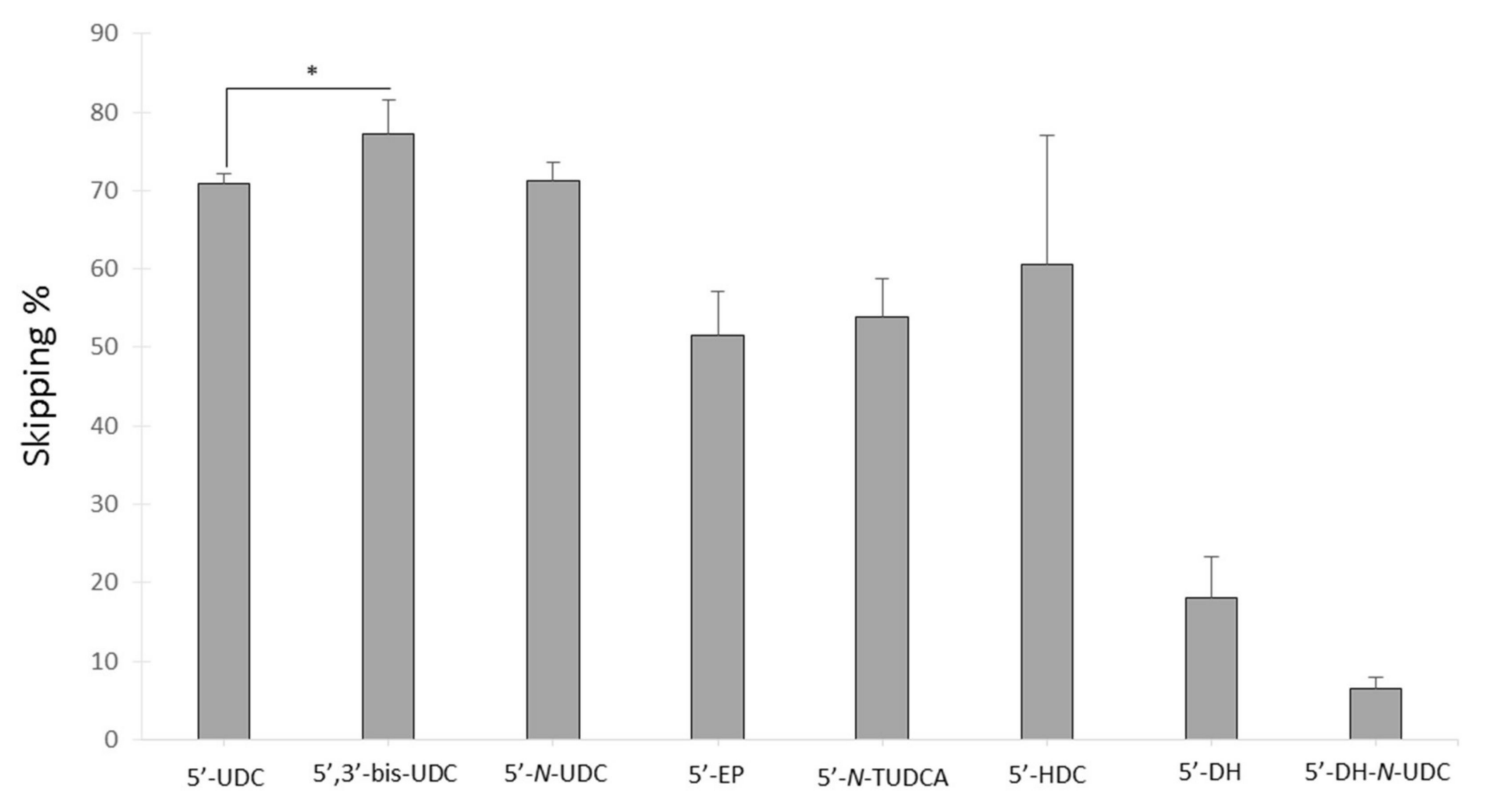
Exon skipping analysis of different lipophilic groups conjugated to ASO 51. Results are expressed as mean ± SEM; *n* = 3 tests per group. * *p* < 0.05 (paired *t*-test with post hoc Mann–Whitney test).

To further study and understand the effect of bile acids and other lipophilic compounds bound to ASO 51, we transfected the conjugated ASO listed in Table S1 in myotubes as explained before (Figure 5).

Conjugating ASO 51 with two UDC group at the 5**′** and 3**′** ends gave the highest skipping percentage when compared to 5**′**-UDC-ASO 51 and 5**′**-*N*-UDC-ASO 51 which were the two others best ASO in this series (average skipping efficiency 77, 70, and 71%, respectively) indicating a potentiation effect of the second UDC group. Linking UDC by tail (as in the case of 5**′**-UDC) or by head (as in 5**′**-*N*-UDC) to the 5**′**-end of ASO 51 did not influence the skipping efficiency. Groups with an increased lipophilicity (EP, DH, and DH-*N*-UDC) in respect to UDC gave the worst skipping efficiencies probably indicating a reduced ability to separate from the cell membranes [26]. Additionally, DH-*N*-UDC has the lowest *T*m reflecting a reduced affinity to its target and possibly contributing to the low skipping efficiency. Altogether, these results point that different and specific mechanisms apart from steric hindrance that can explain the increased skipping efficiency of the conjugated ASO have to be further investigated. In fact, the different conjugates have similar size, with EP being smaller but more efficient than DH.

#### 2.3.4. Gymnotic Delivery of Selected Conjugated ASO 51

**Figure 6:**
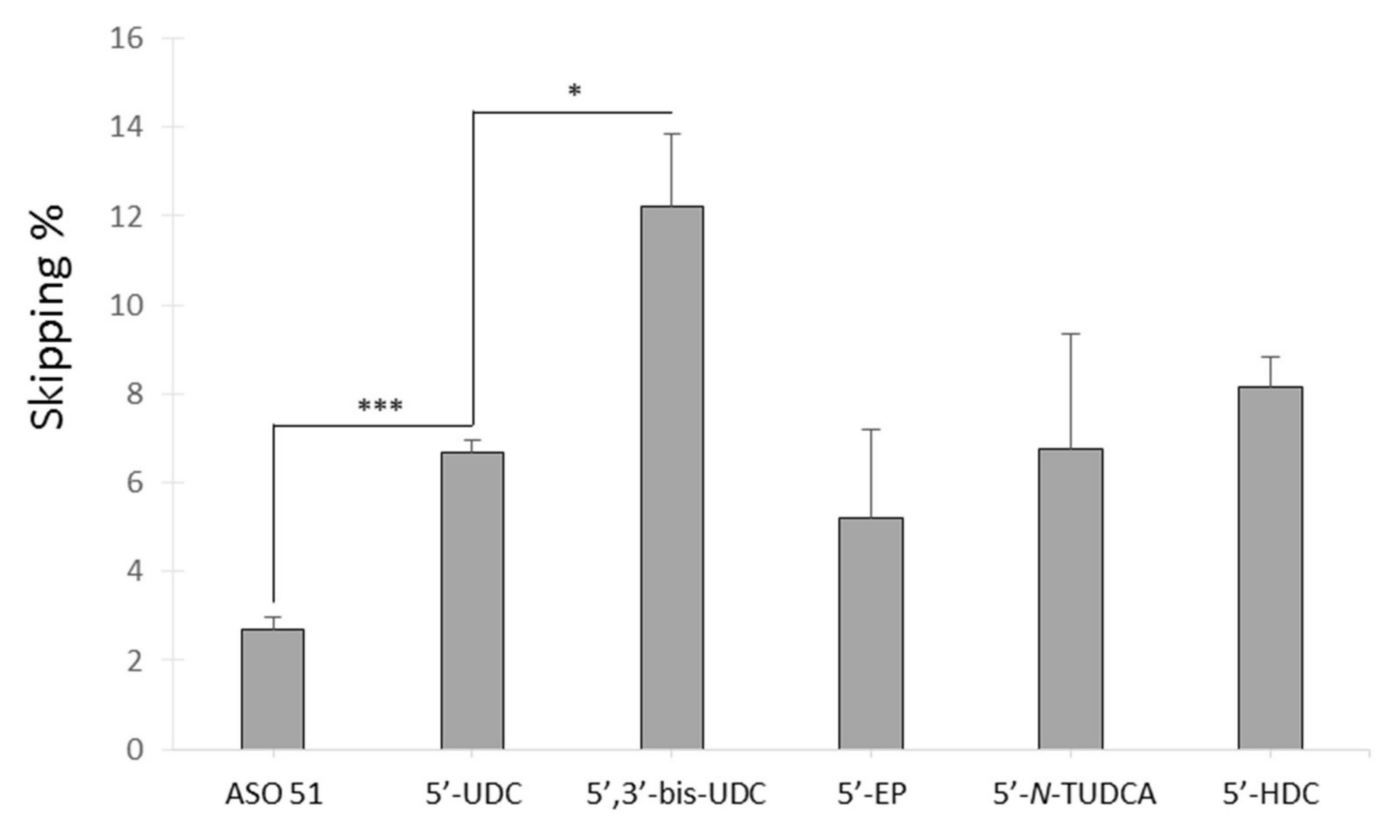
Gymnotic delivery of selected conjugated ASO. Results are expressed as mean ± SEM; *n* = 3 tests per group. *** *p* < 0.001 and * *p* < 0.05 (paired *t*-test with post hoc Mann–Whitney test).

All the ASO with a skipping efficiency threshold higher than the 40% have been investigated for their ability to enter myotubes without the aid of the transfection reagent. This was performed by diluting ASO to a final concentration of 25 uM in 24-well plates and performing skipping analysis 48 h after ASO addition (Figure 6).

Again, in this set of experiments the 5′,3′-bis-UDC-ASO 51 gave the highest skipping efficiencies followed by 5′-HDC and 5′-UDC (average skipping 12, 8, and 6%, respectively). ASO-51 was used as control and resulted in 2.7% of average skipping efficiencies. Apart from 5′,3′-bis-UDC, all the other oligonucleotides did not result significantly different in terms of exon skipping efficiencies with gymnotic delivery when compared to 5′-UDC.

To better understand the mechanism behind the improved uptake of 5′-UDC and 5′-3′-bis-UDC when compared to unconjugated ASO 51, PCS, and TEM analyses were conducted. Indeed, the possible formation of nanoparticles by these oligonucleotides was speculated since it represents an essential property necessary to activate receptor mediated uptake of ASO [44].

### 2.4. Lipophilic-Modified ASO 51 Spontaneously Formed Nanoparticle Aggregates in Acqueous Media

**Figure 7:**
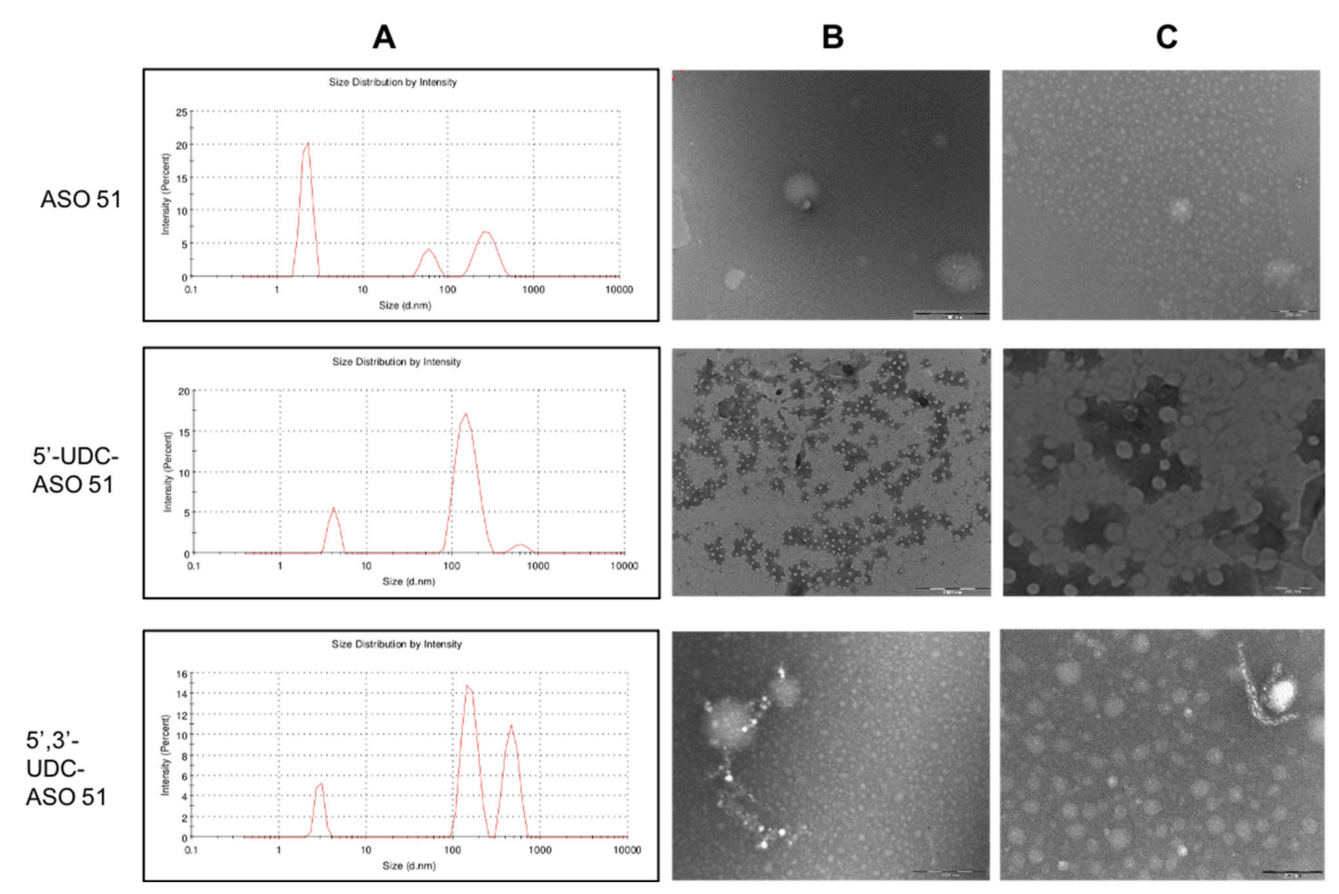
Physicochemical properties of ASO 51, 5′-UDC-ASO 51 and 5′,3′-bis-UDC-ASO 51. (**A**): Size distribution by intensity detected by PCS; (**B**): Negatively stained transmission electron micrographs (scale bar: 1000 nm); (**C**): Magnifications of negatively stained transmission electron micrographs (scale bar: 200 nm).

It is well known that amphiphilic molecules when poured in water or aqueous media rearrange themselves leading to supramolecular aggregates, e.g., micelles or nanoparticles. Starting from this concept the size of the spontaneously formed aggregates was measured by mean of Photon Correlation Spectroscopy (PCS). Figure 7A reports the distribution profiles results of ASO 51, 5′-UDC-ASO 51, and 5′,3′-bis-UDC-ASO 51.

The analysed compounds showed multiple peaks indicating a polydisperse population being in agreement with a possible rearrangement of the compound into aggregates. Particularly, the different populations are ascribable to the single ASO and to its self-assembled aggregates. Moreover, the ability in producing supramolecular nanoparticles was related to the lipophilic moiety conjugated to ASO 51. Indeed, as displayed in Figure 7A the naked ASO 51 is characterized by one intense population around 2 nm (first peak), corresponding to the single entity. The second and third smaller peaks refer to spontaneously formed aggregates as clearly evidenced by TEM images. Overall, the naked ASO 51 is characterized by the smallest dispersed population.

In the case of conjugated ASO 51, different patterns have been obtained. Indeed, for 5′-UDC-ASO 51, the main population was observed around 150 nm (second peak). Possibly, the presence of the lipid conjugate chains led to a rearrangement in nanoparticles, confirming the tendency to form greater aggregates [45]. This behaviour was also appreciable in the case of 5′,3′-bis-UDC-ASO 51 where along with a population around 150 nm, a second one larger than 200 nm was detected (Figure 7A, second and third peak). TEM imaging of ASO 51 samples in aqueous media are in agreement with PCS analysis (Figure 7B,C). For example, 5′-UDC-ASO 51 showed small spherical objects ranging from approximately 40 to 70 nm, whereas in the case of 5′,3′-bis-UDC-ASO 51, greater spherical aggregates other than 40–100 nm were observed in the same sample.

## 3. Materials and Methods

### 3.1. Chemistry

Dry solvents were stored on molecular sieves EZ DRY moisture trap (EMP biotech GmbH, Berlin, Germany); reactions have been monitored by thin layer chromatography with silica gel 60 F245 (Merk Life Science S.r.l., Milano, Italy), spots were visualized with the aid of a phosphomolybdic acid solution. Purification by flash chromatography was executed under nitrogen pressure with silica flash 230–400 mesh (Merk Life Science S.r.l., Milano, Italy).

Oligonucleotides were synthesized using ÄKTA oligopilot 10 PLUS (GE Healthcare, Milan, Italy) controlled via UNICORN software. Commercially available 2**′**-OMe phosphoramidites (DMT-2**′**-OMe-rU-phosphoramidite, DMT-2**′**-OMe-rA-(bz)-phosphoramidite, DMT-2**′**-OMe-rG-(ibu)-phosphoramidite, DMT-2**′**-OMe-rC-(ac)-phosphoramidite and ssH-Linker^TM^ derived from ChemGenes Corporation (Wilmington, MA, USA). Commercially available solid support Primer Support^TM^ 5G Unylinker 350 derived from GE Healthcare (GE Healthcare, Milano, Italy). Oligonucleotides were purified on ÄKTA purifier with a Resource RPC 3 mL column. Ionic exchange was executed on HiTrap Capto S column. Lyophilization was carried out with Labconco FreeZone 1 connected to the centrifuge Christ RVC 2-18 CDplus.

^1^H-NMR (400 MHz) and ^13^C-NMR (101 MHz) were registered in the stated solvent on Varian Mercury Plus 400 (Varian, Palo Alto, CA, USA). ESI-MS spectra were obtained on spectrometer Thermo Finnigan LCQ Duo Ion Trap (Thermo Finnigan LLC, CA, USA) spectrophotometric analysis were executed with Varian CARY 100 Bio instrument (Agilent Technologies, Santa Clara, CA, USA) in the stated solvent.

#### 3.1.1. General Procedure for the Synthesis of NHS-Esters

NHS-esters **1**–**4** have been synthesized following our procedure previously reported for NHS-ester **1** [27].

##### Characterization of NHS-Ester of HDCA **2**

Purified by crystallization from AcOEt, white amorphous solid, yield 79%.

MS (ESI, *m/z*, ES^+^): calculated for [C_28_H_43_NO_6_ + Na]^+^ 512.64; found 512.40; calculated for [2∙C_28_H_43_NO_6_ + H]^+^ 980.31; found 979.27. MS (ESI, ES^−^): calculated for C_28_H_43_NO_6_ 489.65; found 488.47 [M-H]^−^, 977.27 [2M-H]^−^.

^1^H-NMR (400 MHz, CDCl_3_): *δ* = 4.08–3.99 (m, 1H, 6β-H), 3.66–3.55 (m, 1H, 3β-H), 2.83 (d, *J* = 4.4 Hz, 4H), 2.69–2.60 (m, 1H), 2.57–2.46 (m, 1H), 1.99–0.99 (m, 24H), 0.94 (d, *J* = 6.3 Hz, 3H), 0.89 (s, 3H), 0.64 (s, 3H).

^13^C-NMR (101 MHz, CDCl_3_): *δ* = 169.21, 169.07, 71.44, 71.35, 55.66, 54.75, 45.77, 43.74, 42.40, 40.08, 39.13, 37.24, 36.79, 35.04, 34.90, 34.06, 30.61, 30.29, 28.55, 28.00, 26.85, 25.58, 25.38, 23.37, 21.15, 18.28, 12.10, 8.60.

##### Characterization of NHS-Ester of DHA **3**

Flash chromatography (Cyclohexane/AcOEt 4:1), orange syrup, yield 78%.

MS (ESI, *m/z*, ES^+^): calculated for C_26_H_35_NO_4_ 425.57; found 425.47 [M+H]^+^.

^1^H-NMR (400 MHz, CDCl_3_): *δ* = 5.59–5.21 (m, 12H), 2.93–2.74 (m, 14H,), 2.67 (t, *J* = 7.1 Hz, 2H), 2.50 (q, *J* = 7.2 Hz, 2H), 2.07 (quint, *J* = 7.5 Hz, 2H), 0.97 (t, *J* = 7.5 Hz, 3H).

^13^C-NMR (101 MHz, CDCl_3_): *δ* = 169.08, 168.06, 132.02, 130.34, 128.53, 128.42, 128.24, 128.06, 127.87, 127.78, 126.99, 126.46, 30.91, 25.57, 22.33, 20.55, 14.27.

##### Characterization of NHS-Ester of EPA **4**

Flash chromatography (Cyclohexane/AcOEt 4:1), orange syrup, yield 77%.

MS (ESI, *m/z*, ES^+^): calculated for C_24_H_33_NO_4_ 399.53; found 400.80 [M+H]^+^.

^1^H-NMR (400 MHz, CDCl_3_): δ = 5.48–5.26 (m, 10H), 2.89–2.75 (m, 12H), 2.61 (t, *J* = 7.5 Hz, 2H), 2.19 (q, *J* = 7.4 Hz, 2H, 4-CH_2_), 2.07 (quint, *J* = 6.8 Hz, 2H, 3-CH_2_), 1.82 (quint, *J* = 7.5 Hz, 2H), 0.96 (t, *J* = 7.5 Hz, 3H).

^13^C-NMR (101 MHz, CDCl_3_): δ = 169.24, 169.10, 71.56, 68.03, 56.09, 55.77, 48.35, 42.88, 39.89, 39.75, 35.92, 35.52, 35.12, 34.98, 34.80, 30.54, 30.20, 29.17, 28.00, 24.16, 23.47, 20.72, 18.15, 12.00.

##### 3.1.2. Synthesis of 3-α-Amino UDCMe **6**

To a solution of 3α-N_3_-UDC-OMe **5** (1.043 mmol) and HCOONH_4_ (10.430 mmol) in AcOEt/MeOH 1:1 (10 mL), Pd/C (2.086 mmol) in MeOH (5 mL) was added slowly. The mixture was stirred at 70 °C for 18 h. Pd/C was removed by filtration. The solvent was removed under reduced pressure and the residue was extracted with CH_2_Cl_2_ (15 mL) and washed with brine (10 mL). The extract was dried over Na_2_SO_4_ and CH_2_Cl_2_ was removed under reduced pressure to obtain **6** as a crude solid. Additional purification was not required. White amorphous solid, yield 78%.

MS (ESI, *m/z*, ES^+^): calculated for C_25_H_43_NO_3_ 405.62; found 406.33 [M+H]^+^, 811.27 [2M+H]^+^, 1215.87 [3M+H]^+^. MS (ESI, *m*/*z*, ES^−^): found 404.40 [M-H]^−^, 805.07 [2M-H]^−^.

^1^H NMR (400 MHz, CDCl_3_): *δ* = 3.62 (s, 3H, OMe), 3.59–3.50 (m, 1H, 7α-H), 2.64 (bs, 1H, 3β-H), 2.37–2.27 (m, 1H), 2.23–2.13 (m, 1H), 1.99–0.85 (m, 30H), 0.64 (s, 3H).

^13^C NMR (101 MHz, CDCl_3_): *δ* = 174.66, 70.93, 55.83, 54.93, 51.45, 51.24, 43.73, 43.65, 42.89, 40.15, 39.23, 38.36, 37.19, 35.66, 35.32, 34.11, 31.10, 31.01, 28.62, 26.93, 23.63, 21.16, 18.35, 12.11.

#### 3.1.3. Synthesis of NHS Ester **8**

To a solution of **6** (0.678 mmol) in pyridine (4 mL), succinic anhydride (3.390 mmol) and catalytic DMAP were added. The mixture was stirred at 115 °C for 18 h, then cooled at r.t., diluted with AcOEt (15 mL) and washed with aqueous HCl 5% (3 × 5 mL) and H_2_O (5 mL). The extract was dried over Na_2_SO_4_ and the solvent was removed in vacuo to obtain a crude solid. The crude product was used without further purification in the subsequent reaction of activation of the carboxylic acid with NHS (see “General Procedure for the Synthesis of NHS-Esters”). Flash chromatography (AcOEt/cyclohexane 5:1), white amorphous solid, yield 39% in two steps.

MS (ESI, *m*/*z*, ES^+^): calculated for C_33_H_50_N_2_O_8_ 602.77; found 625.27 [M+Na]^+^, 1227.13 [2M+Na]^+^. MS (ESI, ES^−^): found 601.47 [M-H]^−^, 1238.93 [2M+Cl]^−^.

^1^H-NMR (400 MHz, CDCl_3_): *δ* = 5.66 (d, *J* = 7.9 Hz, 1H), 3.77–3.67 (m, 1H, 7α-H), 3.65 (s, 3H, OMe), 3.57–3.41 (m, 1H, 3β-H), 2.96 (t, *J* = 7.1 Hz, 2H), 2.85 (s, 4H), 2.53 (t, *J* = 7.1 Hz), 2.40–2.29 (m, 1H), 2.26–2.16 (m, 1H), 2.07–0.99 (m, H), 0.94 (s, 3H), 0.91 (d, *J* = 6.4 Hz, 3H), 0.66 (s, 3H).

^13^C NMR (101 MHz, CDCl_3_): *δ* = 174.72, 169.11, 168.98, 168.16, 71.37, 55.84, 55.03, 51.51, 50.43, 49.47, 43.71, 42.74, 40.17, 39.26, 36.71, 35.38, 35.28, 34.30, 34.04, 32.88, 31.09, 31.04, 28.60, 27.52, 27.04, 26.88, 25.57, 25.18, 24.49, 23.52, 21.14, 18.37, 12.11.

#### 3.1.4. Synthesis of Ester **9**

To a solution of **3** (1.752 mmol) in DMF (10 mL), 6 (1.752 mmol), and DIPEA (3.504 mmol; 491 µL) were added. The mixture was stirred at 25 °C for 18 h, then 10 mL of aqueous HCl at pH=5 were added. The mixture was extracted with CH_2_Cl_2_ (30 mL) and washed with aqueous NaHCO_3_ (3 × 10 mL). The extract was dried over Na_2_SO_4_ and CH_2_Cl_2_ was removed under reduced pressure to obtain crude **9**. Flash chromatography (AcOEt/cyclohexane 1:1), afforded **9** as a light-yellow amorphous solid, in 65% yield.

MS (ESI, *m*/*z*, ES^+^): calculated for C_47_H_73_NO_4_ 716.10; found 479.50 [2M+3H]^3+^, 503.37 [2M+3Na]^3+^.

^1^H NMR (400 MHz, CDCl_3_): *δ* = 5.46–5.23 (m, 12H), 3.71 (bs, 1H, 3β-H), 3.66 (s, 3H), 3.57–3.48 (m, 1H, 7α-H), 2.89–2.73 (m, 10H), 2.45–2.29 (m, 3H), 2.26–2.14 (m, 3H), 2.12–0.83 (m, 35H), 0.67 (s, 3H).

^13^C NMR (101 MHz, CDCl_3_): *δ* = 174.72, 171.43, 132.04, 129.23, 128.56, 128.26, 128.06, 127.84, 126.97, 71.34, 55.84, 54.99, 51.54, 49.14, 43.73, 42.76, 40.15, 39.28, 36.66, 35.43, 35.26, 34.63, 34.05, 31.06, 31.01, 28.60, 27.81, 26.87, 25.63, 25.54, 24.89, 23.55, 21.13, 20.56, 18.37, 14.29, 12.11.

#### 3.1.5. Synthesis of NHS-Ester **10**

To a solution of **9** (0.304 mmol) in MeOH (2.5 mL), an aqueous solution of LiOH 1.5M (3.04 mmol) was added. The mixture was stirred at 25 °C for 24 h, then, aqueous HCl 5% was added until pH = 4. The mixture was extracted with AcOEt (3 × 8 mL), the organic phase dried over Na_2_SO_4_ and the solvent was removed in vacuo to obtain a crude solid. The crude product was used without further purification for the synthesis of NHS-ester 10 (see “General Procedure for the Synthesis of NHS-Esters”).

Flash chromatography (AcOEt/cyclohexane 2:1), light-yellow amorphous solid, 43% overall yield.

MS (ESI, *m*/*z*, ES^+^): calculated for C_50_H_74_N_2_O_6_ 799.15; found 799.93 [M+H]^+^.

^1^H NMR (400 MHz, CDCl_3_): *δ* = 5.46–5.25 (m, 12H), 3.79–3.60 (m, 1H, 3β-H), 3.57–3.39 (m, 1H, 7α-H), 2.91–2.76 (m, 14H), 2.70–2.60 (m, 3H), 2.57–2.47 (m, 3H), 2.39 (dd, *J* = 13.9, 7.4 Hz, 3H), 2.28–0.84 (m, 32H), 0.69 (s, 3H).

^13^C NMR (101 MHz, CDCl_3_): *δ* = 171.42, 169.22, 169.07, 132.04, 129.24, 128.57, 128.27, 128.08, 127.85, 126.99, 71.31, 55.82, 54.86, 49.14, 43.79, 43.71, 42.76, 40.15, 39.25, 36.71, 35.44, 35.07, 34.64, 34.05, 32.90, 32.21, 30.63, 28.58, 27.99, 27.81, 26.84, 25.59, 25.15, 24.74, 24.44, 24.11, 23.55, 23.49, 21.13, 20.56, 18.31, 14.31, 12.10.

#### 3.1.6. Synthesis of 3-α-*N*-BOC UDCA **11**

To a solution of 3-α-Amino UDCMe 6 (1.302 mmol) in THF (6 mL), (BOC)_2_O (3.255 mmol), NaHCO_3_ (1.790 mmol) in H_2_O (4 mL) were added and the resulting mixture was stirred for 24 h at 25 °C, then the solvent removed under vacuo. The residue was diluted with AcOEt (10 mL) and washed with aqueous HCl 5% (10 mL), and then the organic phase concentrated under reduced pressure and filtered on a pad of silica gel (AcOEt/cyclohexane 1:2; 80% yield). To the resulting *N*-BOC derivative (1.00 mmol) in MeOH (5 mL), an aqueous solution of LiOH 1.5M (3.00 mmol) was added. The mixture was stirred at 25 °C for 24 h, then, aqueous HCl 5% was added until pH = 4. The mixture was extracted with AcOEt (3 × 8 mL), the organic phase dried over Na_2_SO_4_ and the solvent was removed in vacuo to obtain crude **11** as an amorphous solid, in 77% yield.

MS (ESI, *m*/*z*, ES+): calculated for C_29_H_49_NO_5_ 491.71; found 514.6 [M+Na]^+^, 983.5 [2M+H]^+^, 1006.1 [2M+Na]^+^, 1496.6 [3M+Na]^+^. MS (ESI, ES^−^): found 490.4 [M-H]^−^, 891.5 [2M-H]^−^, 1472.2 [3M-H]^−^.

^1^H NMR (400 MHz, CD_3_OD) *δ* = 7.36–7.16 (m, 1H), 3.77–3.67 (m, 1H, 7α-H), 3.59–3.34 (m, 1H, 3β-H), 2.40–2.29 (m, 1H), 2.26–2.16 (m, 1H), 2.07–0.99 (m, H),1.42 (s, 9H), 0.94 (s, 3H), 0.91 (d, *J* = 6.4 Hz, 3H), 0.66 (s, 3H).

^13^C NMR (101 MHz, CD_3_OD) *δ* = 175.17, 70.54, 56.05, 55.01, 50.29, 48.35, 48.21, 47.93, 47.78, 47.46, 47.25, 46.97, 46.62, 43.60, 42.52, 40.17, 39.27, 37.25, 35.72, 35.11, 34.57. 33.26, 32.83, 32.55, 31.73, 28.33, 27.04, 25.04, 22.74, 22.37, 21.04, 17.70, 11.29, 7.90.

#### 3.1.7. Synthesis of 3-α-Amino TUDCA **12**

*N*-BOC amino acid **11** (0.234 mmol) in THF (1 mL) was treated with ethylchloroformate (0.257 mmol) and TEA (0.257 mmol) at 0 °C for 60 min generating a mixed anhydride in situ. To the cooled mixture, taurine (0.257 mmol) dissolved in 1 M NaOH aqueous solution (0.3 mL), was added and after warming to 25 °C, the reaction was stirred for additional 24 h. After adjusting the pH to 1 by adding diluted H_2_SO_4_, the resulting 3-α-*N*-BOC-TUDCA was extracted with 1-butanol (3 × 10 mL), then the organic phase was dried over Na_2_SO_4_ and concentrated in vacuo. Finally, the deprotection of BOC-group was carried out by using TFA (1.5 mL) in CH_2_Cl_2_/MeOH 2:1 at 0 °C for 30 min, after which, the removal of solvents under reduced pressure gave *N*-TUCA 12 as amine trifluoroacetate in 52% yield. Crude **12** was used for conjugation with ASO 51 without any purification step.

MS (ESI, *m*/*z*, ES+): calculated for C_26_H_46_N_2_O_5_S 498.72; found 499.4 [M+H]^+^, 521.4 [M+Na]^+^, 997.5 [2M+H]^+^, 1019.5 [2M+Na]^+^, 1496.4 [3M+H]^+^, 1517.6 [3M+Na]^+^. MS (ESI, ES-): found 497.6 [M-H]^−^, 996.5 [2M-H]^−^, 1494.7 [3M-H]^−^.

^1^H NMR (400 MHz, DMSO-*d*_6_) *δ* = 9.64 (t, J = 1.7 Hz, 2H), 5.86–5.59 (m, 12H), 4.36 (t, *J* = 5.7 Hz, 2H), 4.11–3.61 (m, 53H), 3.44 (dt, *J* = 9.3, 6.4 Hz, 4H), 3.38–3.21 (m, 11H), 2.48 (dt, *J* = 3.7, 1.8 Hz, 4H), 2.43–2.28 (m, 27H), 1.93–1.81 (m, 31H), 1.70–1.42 (m, 17H), 1.27 (dd, *J* = 46.2, 34.8 Hz, 149H). 

^13^C-NMR: *δ* = 171.9, 69.0, 60.0, 55.4, 54.5, 50.5, 45.6, 42.9, 42.8, 42.1, 40.0, 38.4, 37.2, 35.4, 34.8, 34.5, 33.6, 32.5, 31.4, 28.0, 26.6, 26.0, 23.1, 20.7, 18.4, 11.9.

#### 3.1.8. General Procedure for Solid Phase Synthesis of Lipophilic-Modified ASO Targeting Human DMD Exon 51

All ASO targeting human DMD exon 51 were synthesized at 7 µmole scale following our protocol for the synthesis of 2′OMe PS oligonucleotides [27]; PO linkage were performed in classical conditions; ssH-Linker^TM^ was inserted at 5*′*-end of ASO after addition of the last nucleotide of sequence, exploiting the phosphoramidite chemistry and the MMT group was removed by using the classical detritylation step.

All conjugations were performed in solid phase. Reaction conditions and characterizations of the purified conjugates by ESI-MS are reported in Table S1.

#### 3.1.9. General Procedure for Oligonucleotide Purifications

All L-ASO conjugates were purified by ion pair HPLC (IP-HPLC) using ion pairing buffer conditions: buffer A: pH8.0 triethylammonium acetate (TEAA) with 5% ACN; buffer B: ACN, working in acetonitrile gradient (15% B in two column volumes, 45% B in four column volumes) and the counterion (TEA^+^) was exchanged with sodium cation by ion exchange chromatography.

#### 3.1.10. UV Melting Studies

The melting temperature (T_m_) of each L-ASO conjugate against its complementary RNA strand was measured as reported in our previous study. Briefly, each L-ASO and its complementary RNA strand (1:1 stoichiometry) were dissolved in 10 mM phosphate buffer NaH_2_PO_4_ (pH 7) containing 150 mM NaCl to a final concentration of 2 μM. The melting curves (absorbance versus temperature) were recorded at 260 nm by applying a heating–cooling–heating cycle in the temperature range of 25–90 °C at a sweep rate of 0.4 °C/min. All experiments were performed in triplicate.

#### 3.1.11. Photon Correlation Spectroscopy (PCS)

The size of ASO aggregates in aqueous solution was measured using a Zetasizer Nano S90 (Malvern Instr., Malvern, UK) equipped with a 5 mW helium neon laser with a wavelength output of 633 nm. Samples were appropriately diluted. Plasticware was cleaned with detergent washing and rinsed twice with milliQ water. Measurements were made at 25 °C at an angle of 90°, run time around 180 s. Data were interpreted by the “CONTIN” method [46].

#### 3.1.12. Transmission Electron Microscopy (TEM) Studies

ASO solutions were prepared in 0.9% saline at 400 μM concentration and analysed using TEM (EM910 by Zeiss, Germany). The samples were prepared by transferring a drop of ASO solution to a carbon-coated copper grid for 15 min and blotted dry, followed by staining for 3 min with 1% phosphotungstic acid. Samples were analysed at 120 kV.

### 3.2. Exon Skipping Studies

Immortalized myoblasts obtained from a patient with exon 52 deletion (Δ52) and kindly provided by Professor Vincent Mouly were maintained in Skeletal Muscle Growth Medium (Promocell, Heidelberg, Germany) and differentiated into myotubes for at least 7 days in Skeletal Muscle Differentiation Medium (Promocell) supplemented with 2% horse serum at 37 °C with 5% CO_2_ incubation. Myotubes were transfected in 24-well plates with either 2 μg of ASO 51 or conjugated ASOs using 4 uL of JetPEI (Polyplus).

Experiments with ASOs with reduced PS content were performed with 1 μg of each ASO and 2 uL of JetPEI.

Gymnotic transfer was performed by diluting 100 mM solutions of ASOs directly into the differentiation medium in 24-well plates to obtain a final concentration of 25 uM.

Total RNA was isolated from myotubes two days after ASO transfection or gymnotic transfer using the RNeasy Kit (Qiagen, Hilden, Germany) and reverse-transcribed by means of a High-Capacity cDNA Reverse Transcription Kit (Applied Biosystems, Foster City, CA, USA), according to the manufacturer’s instructions. Before cDNA synthesis, RNA was treated with DNase I (Roche, Woerden, The Netherlands) and assessed for residual DNA contamination by a 55-cycle PCR.

A total of 28 cycles RT-PCRs were performed with primers designed on exon 50 (DMDex50F CCTGACCTAGCTCCTGGACT) and exon 54 (DMDex54R GTCTGCCACTGGCGGAGGTC) reported in the http://dmd.nl/ (accessed on 9 March 2022) website and checked on a 1% gel. One microliter of the RT-PCR was loaded on high-sensitivity DNA chips (Agilent, Santa Clara, CA, USA) for the quantification of the exon skipping which was performed calculating the ratio of the area of the skipped transcript and the sum of the area of unskipped and skipped transcripts multiplied for 100.

Experiments were performed in triplicates for transfection studies and in duplicate for gymnotic and ASOs with reduced PS content studies due to the limiting amount of synthetized oligos. Significance was evaluated by paired *t*-test with post hoc Mann–Whitney test for *p* values less than 0.05 considered significant.

#### Immunofluorescence Analysis Dystrophin Expression in Myotubes Treated with ASO

In vitro differentiated immortalized Δ52 myoblasts grown on 35 mm u-dishes (Ibidi, Gräfelfing, Germany) were washed with PBS (Sigma-Aldrich, St. Louis, MO, USA), fixed with 4% paraformaldehyde (Sigma-Aldrich) at room temperature for 10 min, and permeabilized with ethanol 75% (Sigma-Aldrich) at room temperature for 1 min. As a blocking solution, 20% fetal bovine serum (Sigma-Aldrich) was used at room temperature for 30 min to reduce secondary antibody background signal. The cells were subsequently incubated overnight at 4 °C with the anti-dystrophin (NCL-DYS2 Novocastra) primary antibody. After incubation, cells were washed with PBS and then incubated with the 594-fluorochrome conjugated secondary antibodies (Thermo Fisher, Waltham, MA, USA) together with DAPI for nucleic acid staining (Ibidi) for 1 hr at room temperature in PBS containing 0.2% Triton X-100. After three successive washings with PBS, dishes were mounted using fluorescent mounting medium (Ibidi) and examined by fluorescence microscopy (DMI6000B, Leica, Milan, Italy).

## 4. Conclusions

In conclusion, it has been obtained a series of conjugates of ASO 51 carrying a lipophilic moiety at 5**′**-end or at both 5**′**- and 3**′**-positions. Three bile acids and two fatty acids were derivatized for the conjugation. The linking of lipophilic moieties to ASO 51 were performed in solid phase and the amide bond was chosen as the coupling chemistry.

The ASO 51 conjugates formed stable duplex with their complementary RNA with the only exception being 5**′**-DH-*N*-UDC carrying a residue of DHA and of UDCA linked together at 5**′**-end.

The effect of variable PS links was evaluated for the 5**′**-UDC-ASO 51 which resulted more efficient in skipping exon 51 when all links were PS and when compared against the ASO 51 3**′**-UDC. The effects of bile acids and lipophilic conjugates on exon skipping were evaluated by transfection and gymnotic delivery overall identifying 5**′**-UDC- and 5**′**,3**′**-bis-UDC-ASO 51 as the more efficient compounds in terms of exon skipping.

Results from TEM and PCS revealed the improved ability of these two conjugated ASO to aggregate into nanoparticles of size compatible with receptor mediated uptake (40 to 150 nm). Unconjugated ASO 51 was mostly detected as disorganized oligonucleotide underlying the property of UDC to form aggregates suitable to cell uptake when conjugated to ASO. These results may explain the higher skipping efficiency for 5**′**-UDC- and 5**′**,3**′**-bis-UDC-ASO 51.

Thus, 5**′**-UDC- and 5**′**,3**′**-bis-UDC-ASO51 could be promising candidates for future experiments in an in vivo model of DMD.

Altogether, the data reported here represent an innovation in the field of antisense oligonucleotides. Although we do not yet know the mechanism, we demonstrated that linking biological moieties to ASO not only improves their uptake by the cells, but also increases their ability to induce exon skipping of several magnitudes when compared to unconjugated ones. Interestingly, these moieties may have important contribution in the biodistribution and in vivo delivery of these ASO, which need to be explored.

## 5. Patents

The work reported in this article is protected by the patent WO 2020/084488 Al 2020.

## Figures and Tables

**Table 1:** The 5′-UDC ASO sequences targeting human DMD exon 51.

Name	Sequence 5′-3′
5′-UDC-ASO 51	U*C*A*A*G*G*A*A*G*A*U*G*G*C*A*U*U*U*C*U
5′-UDC-ASO 51–68% PS	U*C*A*AG*GA*AG*AU*GG*CA*U*U*U*C*U
5′-UDC-ASO 51–53% PS	U*CA*AG*GA*AG*AU*GG*CA*UU*U*C*U
5′-UDC-ASO 51–42% end-PS	U*C*A*A*GGAAGAUGGCAU*U*U*C*U
5′-UDC-ASO 51–42% PS	U*C*AAG*GAA*GAU*GGC*AUUU*C*U
5′-UDC-ASO 51–32% PS	U*C*A*AGGAAGAUGGCAUU*U*C*U

* Corresponds to PS link.

**Table 2:** *T*m values (°C) from UV melting curves (Absorbance 260 nm) relative to RNA/RNA duplex.

Name	*T*m *
ASO 51	71.02
5′-UDC-ASO 51	70.25
5′,3′-bis-UDC-ASO 51	72.02
5′-HDC-ASO 51	69.02
5′-DH-ASO 51	69.04
5′-EP-ASO 51	69.47
5′-*N*-UDC-ASO 51	69.02
5′-*N*-TUDCA-ASO 51	69.02
5′-DH-*N*-UDC-ASO 51	67.50

* Experiments performed in 150 mM NaCl, 10 mM NaH_2_PO_4_ pH 7, *c* = 2 µM.

## Data Availability

Data sharing is not applicable to this article.

## Supplementary Materials

The following supporting information can be downloaded at: https://www.mdpi.com/article/10.3390/ijms23084270/s1.
