# Supplementary material for: Antisense Oligonucleotides Conjugated with Lipophilic Compounds: Synthesis and In Vitro Evaluation of Exon Skipping in Duchenne Muscular Dystrophy
Source: Int J Mol Sci. 2022 Apr 12;23(8):4270. doi: 10.3390/ijms23084270 (PMC9032562; doi:10.3390/ijms23084270)
Supplement: Supplementary file 1 [file ijms-23-04270-s001.zip › ijms-1662139-supplementary.pdf]

Antisense Oligonucleotides Conjugated with Lipophilic Compounds: Synthesis and in Vitro Evaluation of Exon Skipping in Duchenne Muscular Dystrophy

Elena Marchesi[1], Rita Cortesi[2], Lorenzo Preti[2], Paola Rimessi[3], Maddalena Sguizzato[2], Matteo Bovolenta[4,*] and Daniela Perrone[1,*]

[1] Department of Environmental and Prevention Sciences, University of Ferrara, 44121 Ferrara, Italy; mrclne@unife.it

[2] Department of Chemical, Pharmaceutical and Agricultural Sciences, University of Ferrara, 44121 Ferrara, Italy; crt@unife.it (R.C.); prtlnz@unife.it (L.P.) and sgzmdl@unife.it (M.S.)

[3] Unit of Medical Genetics, S. Anna University-Hospital, Ferrara, Italy; rsp@unife.it

[4] Department of Translational Medicine, University of Ferrara, 44121 Ferrara, Italy.

* Correspondence: prd@unife.it (D.P.); bvlmtt@unife.it (M.B.)

Table S1. L-ASO conjugates: reaction conditions for formation of amide bond and ESI-MS analyses

**L (**ASO 51)[a]	**Conditions** [b]	**Efficiency**[c] **(%)** [c]	**Molecular Mass, Da**[d]	
			Calc.[M]	Exp. [M]
5′-UDC	**1** (10 eq.), DIPEA (40 eq.) in DMSO (1 mL), 60 min.	83	7633.66	7631.84
5′-HDC	**2** (10 eq.), DIPEA (40 eq.) in DMSO (1 mL), 60 min.	81	7633.66	7632.64
5′-DH	**3** (10 eq.), DIPEA (40 eq.) in DMSO (1 mL), 60 min	52	7570.35	7566.24
5′-EP	**4** (10 eq.), DIPEA (40 eq.) in DMSO (1 mL), 60 min	53	7544.46	7547.91
5′-*N*-UDC	**8** (10 eq), DIPEA (40 eq.) in DMSO (1 mL), 60 min.	20	7733.70	7735.95
5′-DH-*N*-UDC	**10** (10 eq.), DIPEA (40 eq.) in DMSO (1 mL), 60 min.	15	7943.50	7946.4
5′-*N*-TUDCA	i) Succinic acid (20 eq.), HBTU (20 eq.), DIPEA (40 eq.) in DMF (1 mL), 40 min.; ii) **12** (20 eq.), HBTU (20 eq.), DIPEA (40 eq.) in DMF (1 mL), 40 min.	62	7840.90	7839.01
5′-UDC 68% PS	**1** (10 eq.), DIPEA (40 eq.) in DMSO (1 mL), 60 min.	80	7521.10	7521.08
5′-UDC 53% PS	**1** (10 eq.), DIPEA (40 eq.) in DMSO (1 mL), 60 min.	79	7489.35	7487.60
5′-UDC 42% end-PS	**1** (10 eq.), DIPEA (40 eq.) in DMSO (1 mL), 60 min.	81	7457.25	7457.78
5′-UDC 42% PS	**1** (10 eq.), DIPEA (40 eq.) in DMSO (1 mL), 60 min.	80	7457.25	7459.34
5′-UDC 32% PS	**1** (10 eq.), DIPEA (40 eq.) in DMSO (1 mL), 60 min.	78	7425.15	7427.92
5′,3′-bis-UDC	**1** (10 eq.), DIPEA (40 eq.) in DMSO (1 mL), 60 min.	68	8203.41	8202.64

[a] All synthesized ASO 51 were full-length 2′-OMe and full-length PS unless stated otherwise and contained the following sequence: 5′-UCAAGGAAGAUGGCAUUUCU-3′. [b] All reactions were performed at 7 μmol scale and room temperature by using ssH-ASO 51-S or ss-H-ASO 51 3′-UDC (in the case of 5′,3′-bis-UDC ASO 51). [c] Calculated on UV areas measured at wavelength of 260 nm, of conjugated full-length sequences versus unconjugated ones including failures. [d] According to ESI-MS in the negative ions mode.

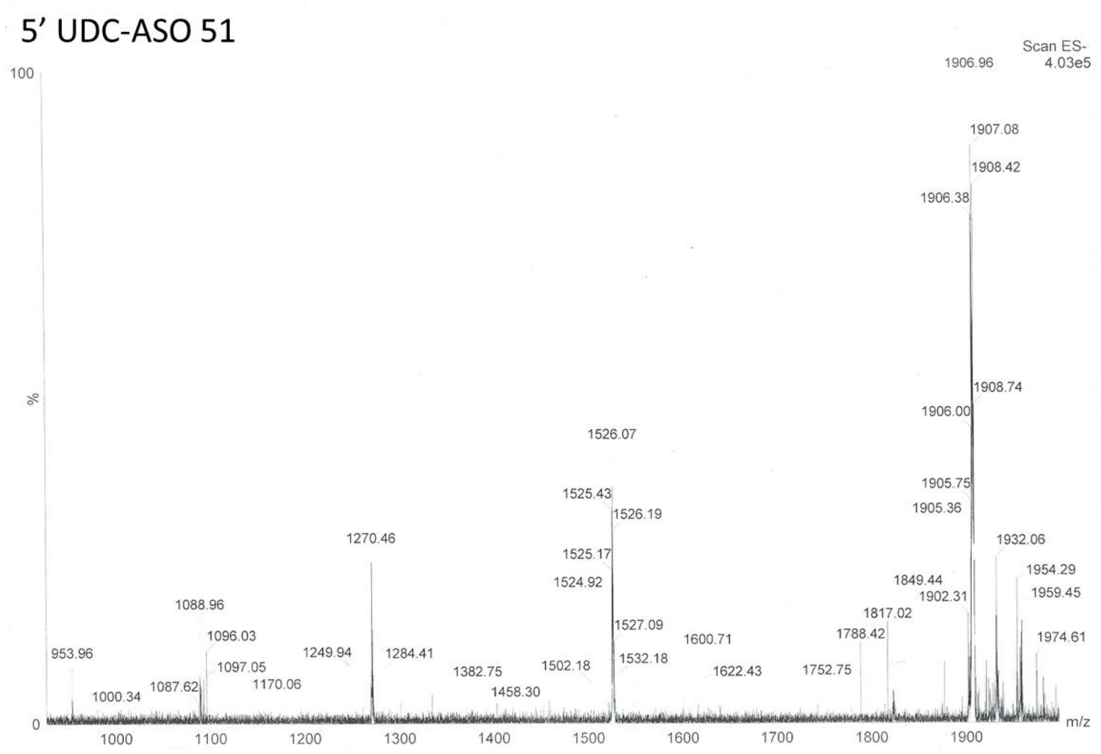

Figure S1: 5′-UDC-ASO 51 ESI-MS spectrum

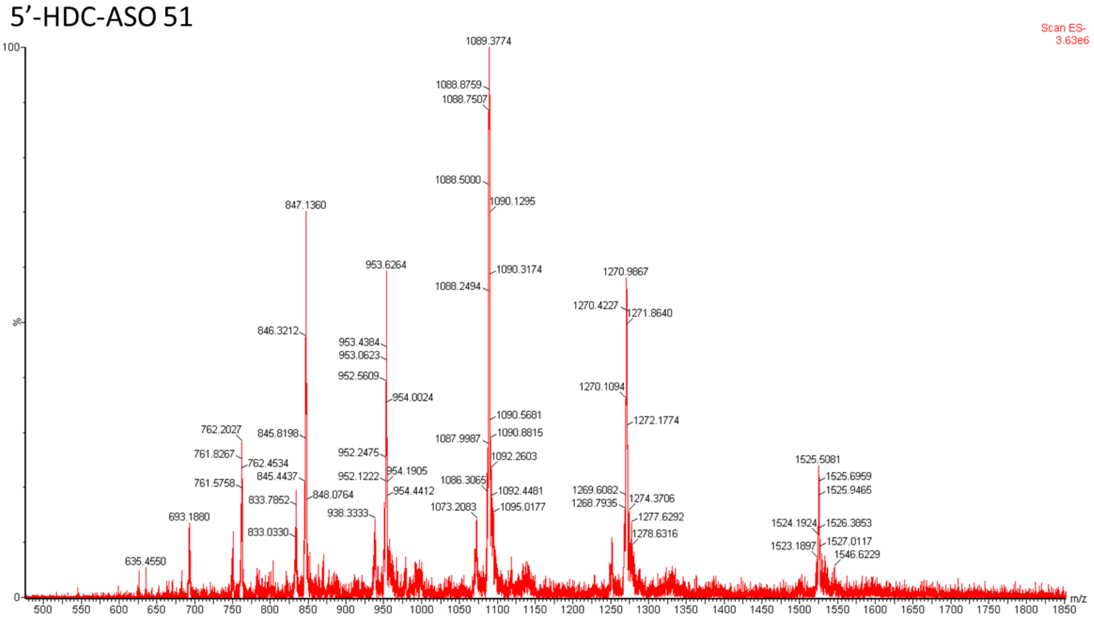

Figure S2: 5′-HDC-ASO 51 ESI-MS spectrum

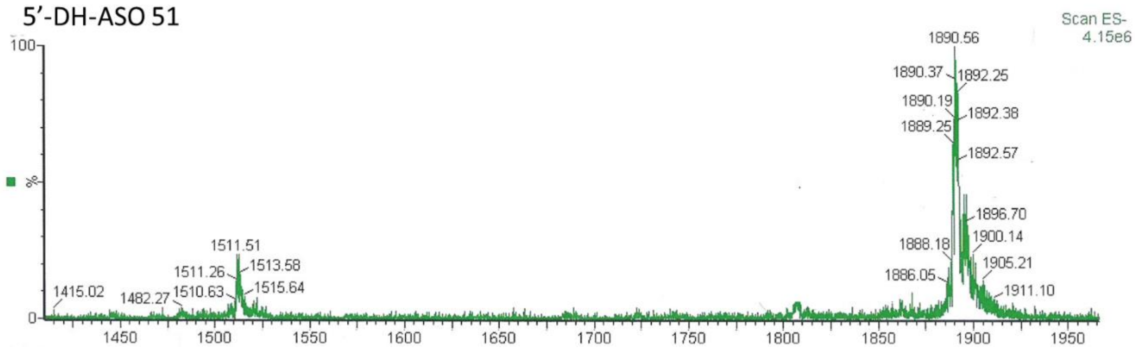

Figure S3: 5′-DH-ASO 51 ESI-MS spectrum

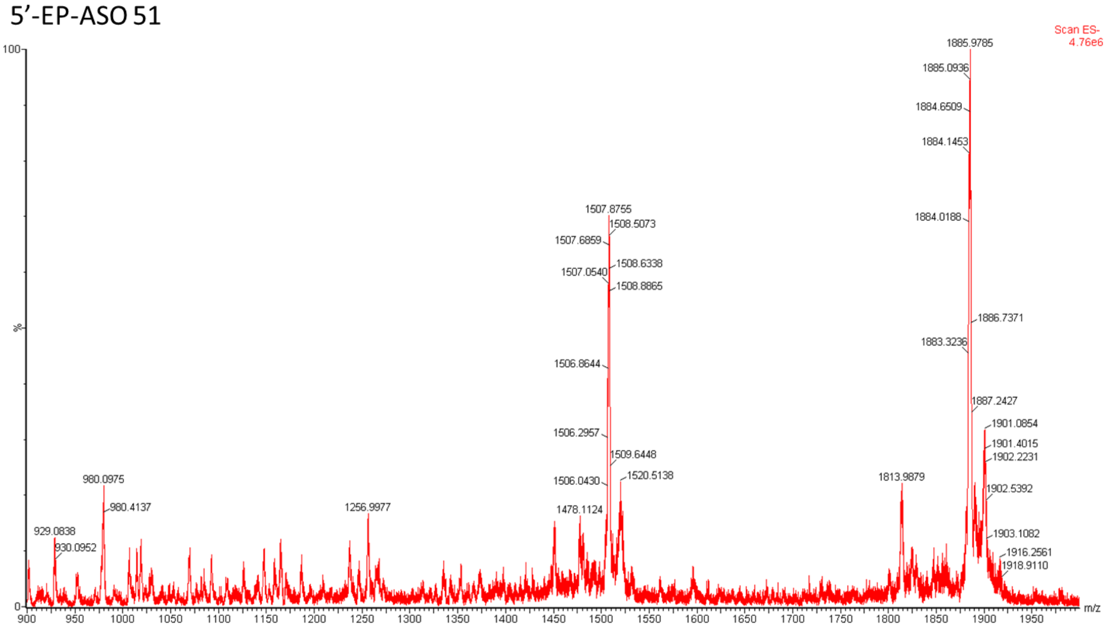

Figure S4: 5′-EP-ASO 51 ESI-MS spectrum

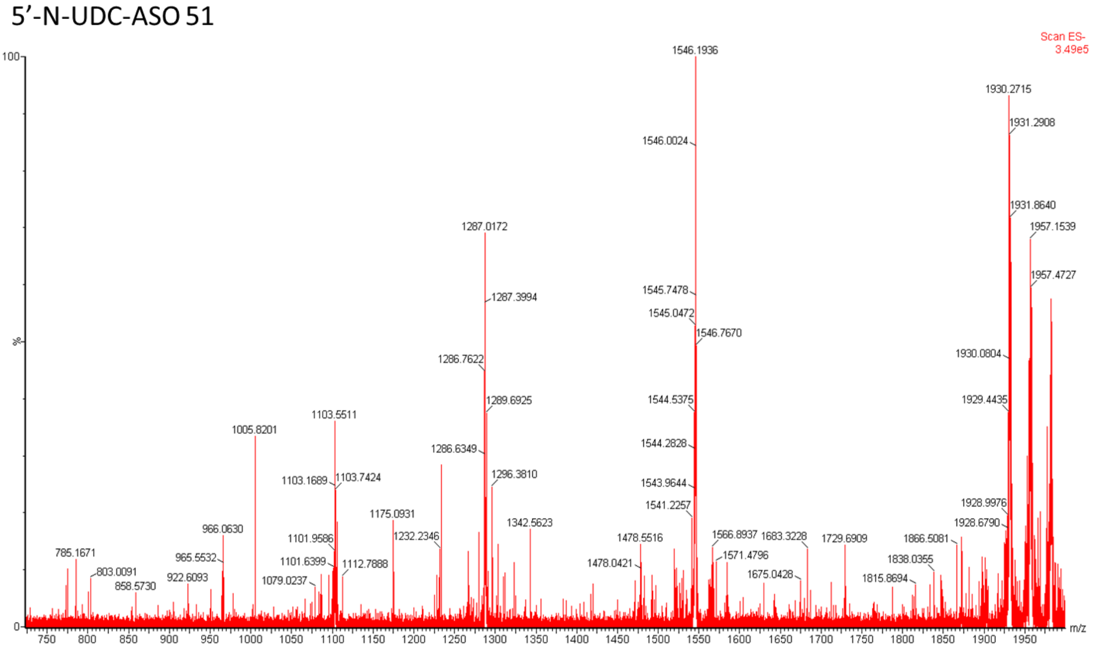

Figure S5: 5′-*N*-UDC-ASO 51 ESI-MS spectrum

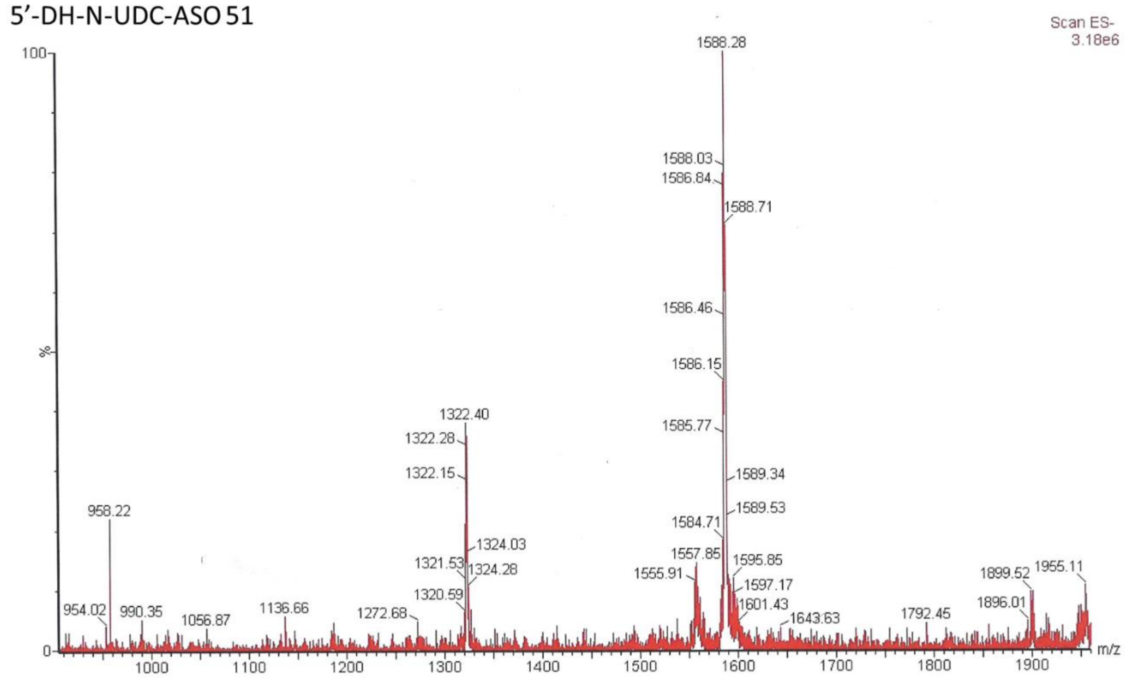

Figure S6: 5′-DH-*N*-UDC-ASO 51 ESI-MS spectrum

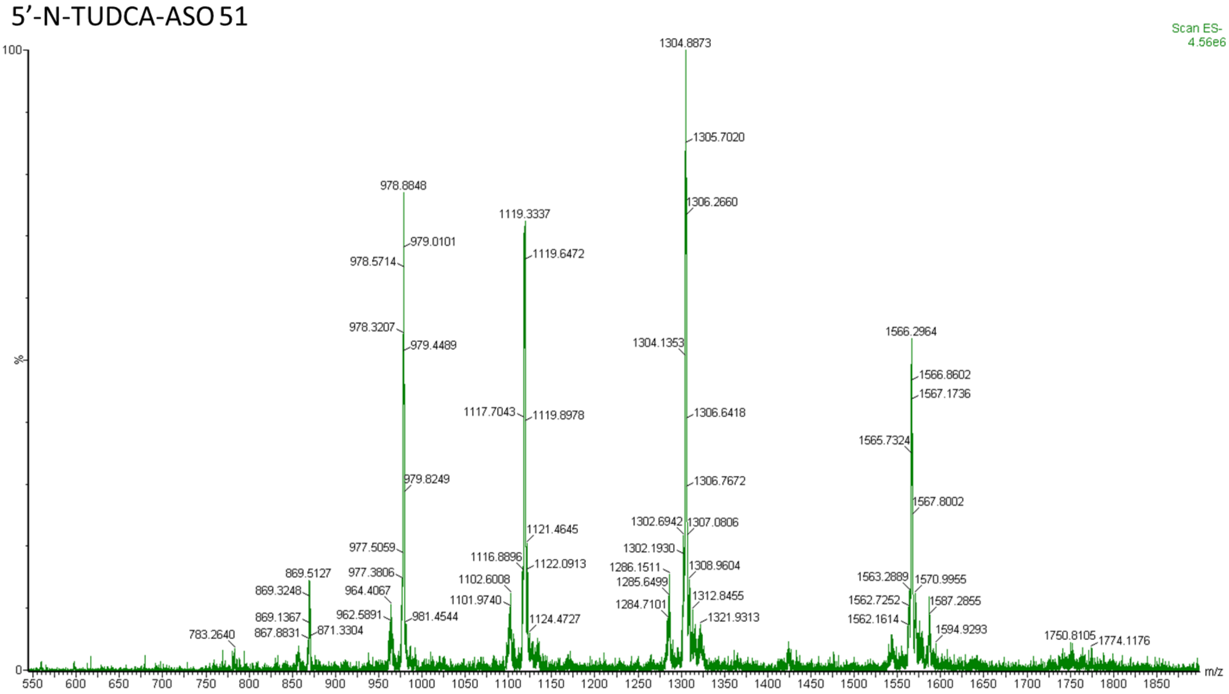

Figure S7: 5′-*N*-TUDCA-ASO 51 ESI-MS spectrum

5′-UDC-ASO 51-68% PS

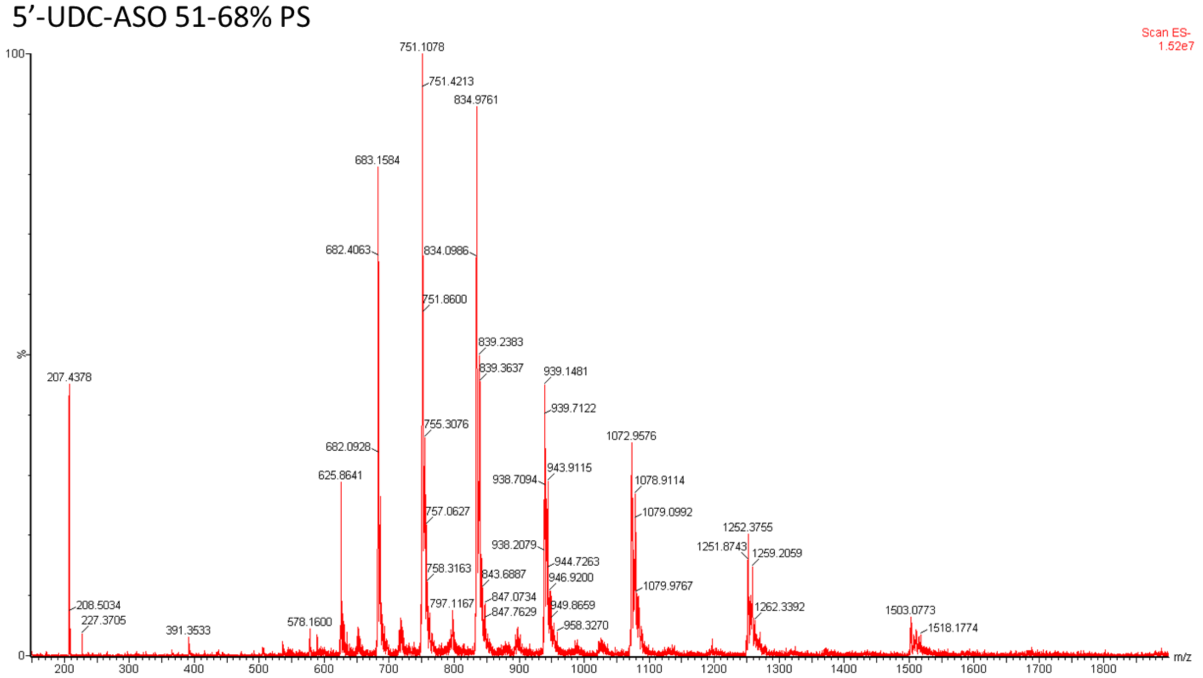

Figure S8: 5′-UDC-ASO 51-68% PS ESI-MS spectrum

5′-UDC-ASO 51-53% PS

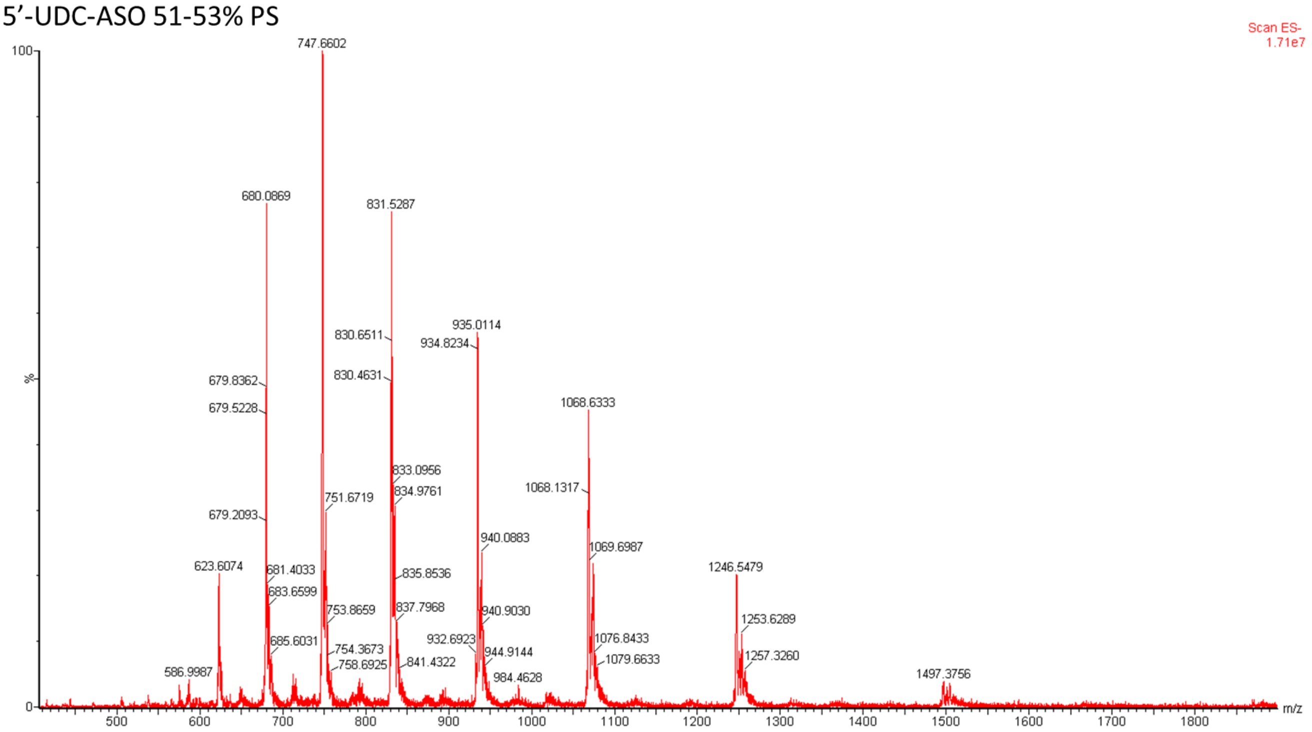

Figure S9: 5′-UDC-ASO 51-53% PS ESI-MS spectrum

5’-UDC-ASO 51-
42% end-PS

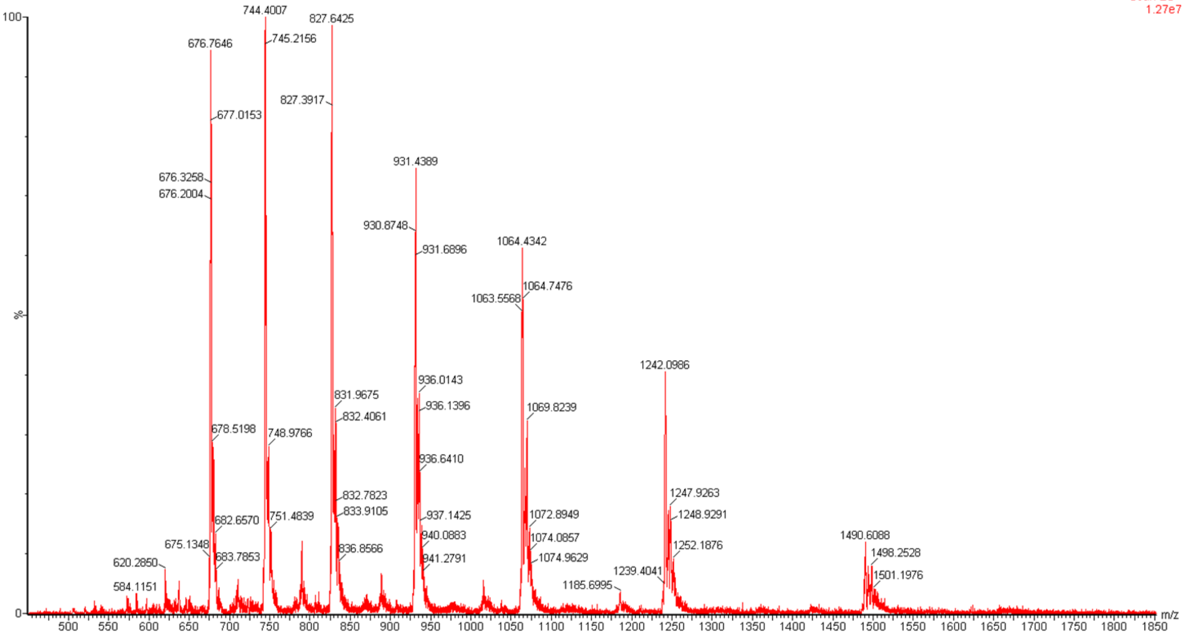

Figure S10: 5’-UDC-ASO 51-42% end-PS ESI-MS spectrum

5’-UDC-ASO 51-
42% PS

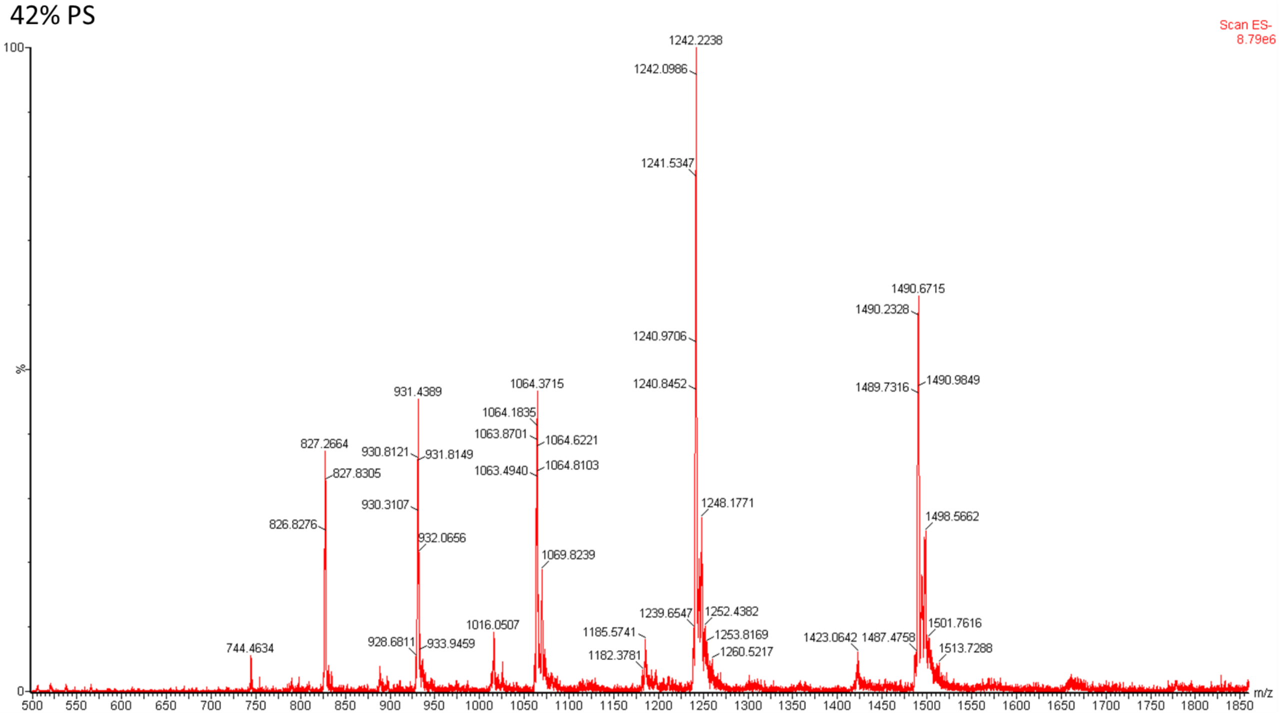

Figure S11: 5’-UDC-ASO 51-42% PS ESI-MS spectrum

5’-UDC-ASO 51-
32% PS

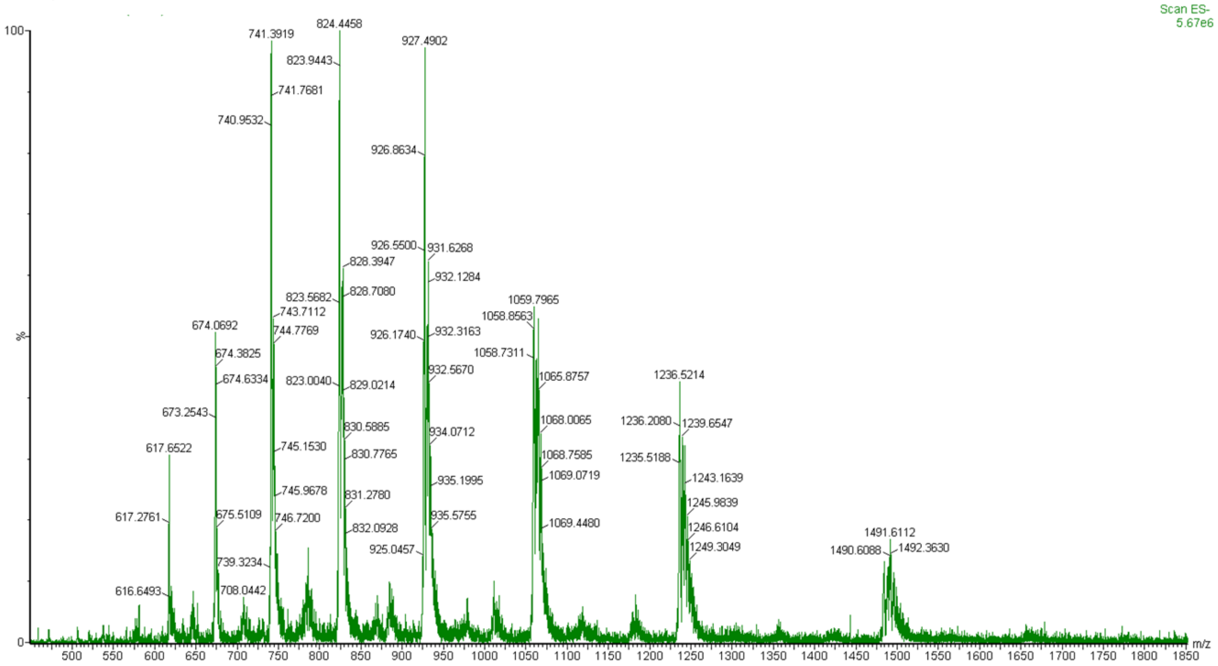

Figure S12: 5’-UDC-ASO 51-32% PS ESI-MS spectrum

5’,3’-bis-UDC-ASO 51

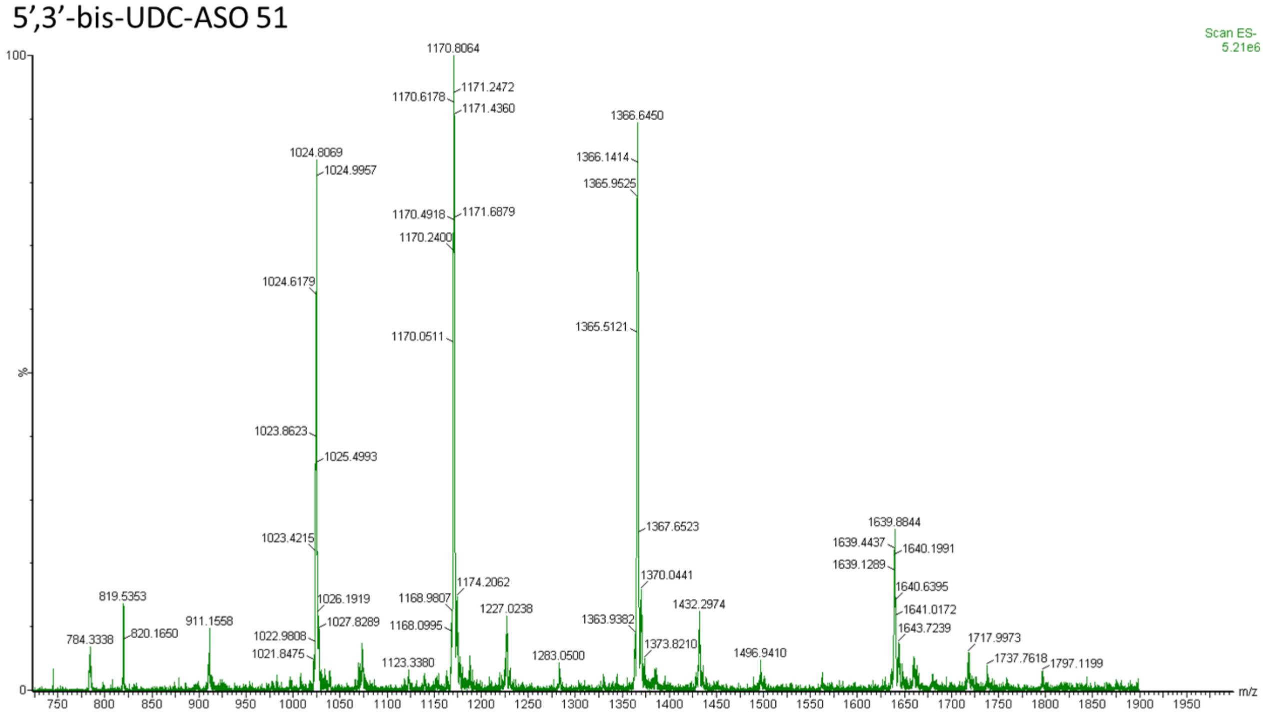

Figure S13: 5′,3′-bis-UDC-ASO 51 ESI-MS spectrum

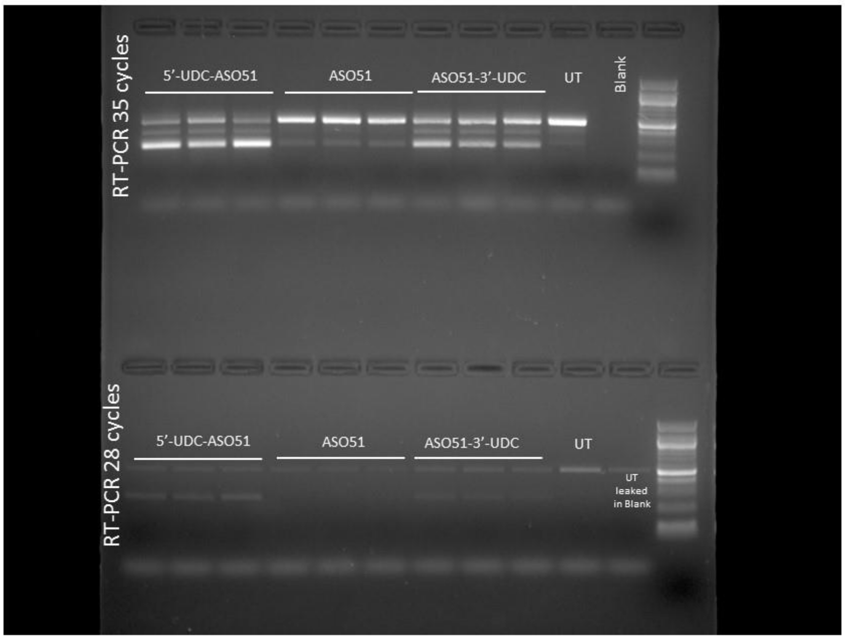

Figure S14: Original images for RT-PCRs performed with primers DMDex50F and DMDex54R
